# Capture and transport of rod-shaped cargo via programmable active particles

**DOI:** 10.1038/s41598-023-42119-9

**Published:** 2023-09-12

**Authors:** Philipp Stengele, Anton Lüders, Peter Nielaba

**Affiliations:** Statistical and Computational Physics, Department of Physics, University of Konstanz, 78457 Konstanz, Germany

**Keywords:** Applied physics, Statistical physics, thermodynamics and nonlinear dynamics, Physics

## Abstract

We study the influence of the cargo shape on the capture and transport process of colloidal rods via swarms of active particles using Brownian dynamics simulations. Starting at random initial conditions, active particles that interact via the Lennard-Jones potential and possess a tuneable speed are utilised to capture passive rods inside a hexagonal cage of individually addressable units. By adjusting the velocity of the individual active particles, the rod can then be transported. To guarantee a successful capture process (with a strong localisation), we find that specific geometric and energetic constraints have to be met; i.e., the length of the rod must approximately be in the vicinity of an odd multiple of the lattice constant of the hexagonal cage, and the Lennard-Jones interaction strength must be in the range of $3.0 \, k_B T$ to $6.0 \, k_B T$. If the cargo aspect ratio gets too large, the subsequent transport of successfully captured rods can fail. For systems where transport is possible, an increase in the cargo aspect ratio decreases the achievable transport velocity. Our work shows that the particle shape must be considered while designing interaction rules to accomplish specific tasks via groups of controllable units.

## Introduction

Using feedback-loop set-ups^1–5^ where colloidal particles can be addressed individually (for instance, via lasers), it is possible to enforce complex “social” interaction rules in soft matter experiments. In these set-ups, the individual velocities and orientations of active particles (APs) can be tuned precisely^6^. Artificial steering signals can, therefore, be computed externally before being inserted back into the system. Examples of phenomena studied with feedback-loop experiments are visual stimuli-induced groups^7^, swirl formation^6^, and cohesion-based clogging of geometric constrictions^8^.

Recently, Yang and Bevan (YB) introduced cargo transport rules for quasi-two-dimensional (quasi-2D) colloid systems with a feedback-loop control^9^. In contrast to other implementations of colloidal cargo transport^10–12^, the YB rules utilise individually controllable APs. Their foundation is to encapsulate a passive spherical cargo particle (CP) within a dense “swarm” of programmable APs that interact via attractive potentials. In more detail, the YB control policy takes advantage of the fact that closely packed spheres generally order in a triangular lattice^13^ by steering the APs to form a hexagonal cage (HC) of controllable units around the CP. The attractions between the individual APs can then stabilise the resulting particle arrangement. After the CP is captured within the HC, it is then transported in a predefined direction^9^. The benefits of the YB rules can be seen in works such as Ref.^14^, where it proved suitable for cargo capture in mazes.

The YB control policy can be split into two interaction rule subsets: The first of these two sets are the capture rules. They modify the magnitude of the APs’ propulsion velocities to form the HC. All APs are assigned to specific locations on a predefined triangular lattice around the CP, and their velocities are increased individually if their orientations randomly point to their corresponding target locations on said lattice. After the CP is successfully captured, the transport rules are engaged. Under these rules, the velocities of APs that randomly point in the predefined transport direction (which we set along the horizontal *x*-axis for simplicity) are increased to propel the system forward. Both subsets of rules only tune the magnitude of the velocity while the APs’ orientations fluctuate exclusively via rotational diffusion.

In their original form, the YB rules are implemented to transport passive spheres of the same size as the spherical APs. This presents a major disadvantage of the control policy: Interaction rules that could, in principle, transport cargo with an arbitrary shape and size are generally much more beneficial. For instance, considering an application in the field of microrobots^15–18^, it is highly advantageous if the same active units could handle a variety of different geometric objects. Furthermore, it was recently shown that a group’s collective motions can strongly depend on the shape of the individuals^19–21^, so there might be additional effects that have to be considered when changing the CP shape.

So far, it is unclear whether the YB rules can be adapted for arbitrarily shaped CPs. We approach this question by studying the capture and transport of colloidal rods with the YB rules via 2D Brownian dynamics (BD) simulations^22–25^ (without hydrodynamic interactions). In our generalisation of the YB rules, we leave a small cavity in the middle of the HC lattice for the CP to fit in. While there are certainly other ways to adapt the policy to rods, we find this approach to be especially promising because it can theoretically be modified for different geometric cargo forms by simply adjusting the shape or size of the cavity.

In our numerical implementation, we model the attractions between the individual APs with the Lennard-Jones (LJ) potential, and we apply the purely repulsive Weeks-Chandler-Andersen (WCA) potential^26^ to the interactions between the APs and the CP (see the "Methods" section). In additional simulations where we replaced the WCA potential with the LJ potential, we also studied the influence of an attraction between CP and the APs. The corresponding results are discussed in Supplementary Sections S1 and S2 of the supporting information (SI).

For reasons of clarity, this work is structured as follows: After a summary of the adjusted YB rules, our analysis is split into two sections highlighting the influence of the CP shape on the individual interaction rule subsets. Firstly, we investigate the capture process starting at random initial conditions. Here, the goal is to reach a stationary state with a strong localisation where the CP is enclosed by the APs and the system’s motion becomes minimal. In the corresponding section, only the capture rules are engaged. Afterwards, we analyse the transport process by trying to precisely move successfully captured CP in a predefined direction via the transport rules.

We find that the YB control policy is sufficient to interact with rod-shaped cargo, which is a stepping stone towards the transport of arbitrarily shaped microparticles via swarms of active units. However, our results show that some geometric and energetic restrictions decide whether the capture and transport processes are successful.

## Interaction rules

**Figure 1:**
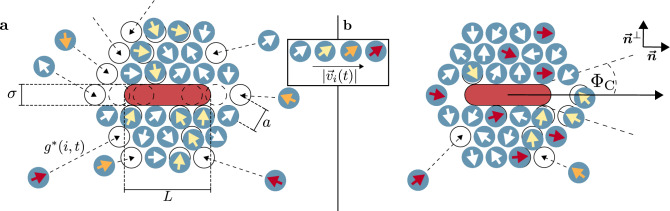
Yang and Bevan rules adjusted for rods. A hexagonal lattice (with lattice constant *a*) of target positions (depicted by the white circles with a black outline) is constructed around a cargo particle with length *L* and diameter $\sigma$. Target positions that would overlap with the rod are removed to form a cavity for the rod (see the circles with a dashed outline). (**a**) To capture the cargo particle, active particles (depicted by the blue circles) are assigned to the target locations. The ideal assignment $g^*(i,t)$ of the target locations is indicated by the dashed black arrows. Depending on the active particles’ orientations and the distances from their assigned targets, individual propulsion velocities $\vec {v}_i(t)$ (shown by the coloured arrows, large velocities are coloured in dark red and small velocities in white) are computed. Particles facing towards their assigned targets obtain a large propulsion velocity, while particles that face away move slowly until they realign via rotational diffusion. (**b**) Cargo transport in a specific direction (indicated by the black arrow and the unit vector $\vec {n}$) can be achieved by setting the propulsion velocity of active particles that face towards the transport direction (with an angular deviation of $\Phi _\mathrm{C}$) to the maximum value. The propulsion velocity of the remaining particles is computed according to the capture rules. The unit vector $\vec {n}^{\perp }$ is perpendicular to the transport direction.

In our simulations, we model the passive rods as spherocylinders (see Fig. 1). A two-dimensional spherocylinder consists of a rectangular middle section enclosed by two semicircular caps. It can be characterised by its length *L*, its width or “diameter” $\sigma$ and the dimensionless aspect ratio $q = L / \sigma$. Identically to YB’s work^9^, the APs are quasi-2D spheres. Throughout our work, they possess the same diameter $\sigma$ as the CP.

To form the HC, first, a hexagonal lattice of target positions $\{ \vec {r}_{\text {T},i}(t) \}$ is constructed around the current CP centre position. These positions are represented by the white circles with a black outline in Fig. 1a. The corresponding lattice constant *a* is set to the distance corresponding to the minimum of the LJ potential (i.e., $a = 2^{1/6} \, \sigma$). This results in a stabilisation of the HC based on the individual AP attractions^9^, which are characterised by the interaction strength $\varepsilon$ (see the "Methods" section). This parameter $\varepsilon$ (that captures the physical attractions between the APs) scales the LJ potential relative to the thermal energy and is, therefore, closely related to the “stiffness” and the total potential energy of the system. There are multiple ways to implement variable AP pair interactions in experimental setups reaching from effective depletion forces (as discussed in Ref.^9^) to cohesion based on artificial interactions (see Ref.^8^). In Supplementary Section S3 of the SI, we briefly discuss some examples of colloidal interactions that can be tuned in experiments.

To enclose a spherical CP with a symmetric HC with *m* layers, $3m^2 - 3m$ target positions are needed, (which is based on the centred hexagonal number adjusted for the CP in the middle). Due to the elongation of the spherocylinder-shaped CPs, target positions in the middle of the lattice have to be removed to form a cavity for the CP. Starting at the formation needed to capture a spherical CP, we remove all target positions that would overlap with a spherocylinder that is positioned in the centre of the lattice and parallel to the predefined transport direction (i.e., parallel to the horizontal *x* axis). This is indicated by the circles with a dashed outline in Fig. 1a. Therefore, for $L \ge 2(a-\sigma / 2)$, only $N = 3m^2 - 3m - 2N'$ with $N' = \lfloor \left\{ L-\left[ 2\left( a - \sigma / 2 \right) \right] \right\} / \left( 2 a \right) \rfloor + 1$ target positions are necessary [here, $\lfloor \cdot \rfloor$ is the floor operator, and $N'$ is set to zero for $L < 2(a-\sigma / 2)$]. Our simulations consist of the CP and *N* APs accordingly. We fixed the numbers of HC layers to $m = 7$ throughout all simulations. Since we vary the CP aspect ratio between $q = 2.36$ and $q = 7.72$, the number of APs is changed in the interval $120\le N\le 124$. We chose this specific value of *m* because it is the minimum number of APs where the cavity is still confined by at least three layers of APs from each side for all studied *q*. This holds the computational costs to a minimum, but the number of layers is large enough that we find it reasonable to say that the rod is fully encased in a HC. The general influence of *m* on the properties of the cargo transport via the YB rules is extensively studied in Ref.^9^, and we assume that it analogously influences the transport of rods (for example, the number of APs increases the mean velocity that can be reached for a fixed $\varepsilon$ during the transport^9^). We also performed some sample simulations analysing the *m* dependence of the system and found that a larger number of layers stabilises the HC during the transport but also increases the time needed for a successful capture process (see Supplementary Section S4 of the SI).

To account for the translational motion of the CP during the capture process (for example, based on diffusion or interactions with the APs), the target position lattice is updated after a control time $\Delta t_{\text {C}}$. At the start of the simulation and after a target position update cycle (i.e., after a time interval of length $\Delta t_{\text {C}}$), each of the APs at the positions $\{ \vec {r}_i(t) \}$ is assigned to one of the target positions. The assignment is chosen so that the total distance all APs have to travel to form the HC becomes minimal^9^. This is done by minimising the cost function

1 $$\begin{aligned} {\mathcal {G}}(g) = \sum _i \left| \vec {r}_i(t) - \vec {r}_{\text {T},g(i,t)}(t) \right| \end{aligned}$$

given in YB’s work^9^. Here, *g*(*i*, *t*) is the index of the assigned target of AP *i* at time *t* according to the hypothetical assignments *g* (the cost function is minimised regarding all possible assignments). The assignment of the APs is implemented via the Hungarian algorithm^27,28^ (a well-established optimisation method that can be applied to solve assignment problems). As this algorithm comes with large computational costs, the control time $\Delta t_{\text {C}}$ is chosen several orders of magnitude larger than the length of a simulation step $\Delta t$ (i.e., the time after which the positions and orientations of the colloids are updated in the simulations). We set the length of a simulation step to $\Delta t = 10^{-5}\,\tau _D$, where $\tau _D = \sigma ^2 / D_{\text {s}}$ is the Brownian time and $D_{\text {s}} = k_B T / (3 \pi \eta \sigma )$ is the translational diffusion constant of a sphere. For the control time $\Delta t_{\text {C}}$, we chose $\Delta t_{\text {C}} = 0.1\,\tau _D$, which fulfils the conditions $\Delta t_{\text {C}} < 1/D^r_{\text {s}} = \tau _D / 3$ and $\Delta t_{\text {C}} < r_{\text {s}}^2/D_{\text {s}} = \tau _D / 4$ that are proposed by YB^9^ and is optimised for computational cost due to the large number of simulations performed in our study and the long total simulation times. Here, $D^r_{\text {s}} = 3 D_{\text {s}} / \sigma ^2$ is the rotational diffusion coefficient of a sphere and $r_{\text {s}} = \sigma / 2$ is the radius of the APs. The assignment of the APs to their target positions is indicated by the dashed arrows in Fig. 1a.

Because the APs can be addressed individually, the magnitude of their propulsion velocities can be adjusted to lead them to their assigned target positions (note once again that the orientations $\{ \vec {e}_i(t) \}$ of the APs change only by rotational diffusion). At each update cycle of the target positions, the magnitude of the propulsion velocity of AP *i* is set to

2 $$\begin{aligned} v_i = \left\{ \begin{array}{ll} \text {min}\left( \frac{d_i}{\Delta t_{\text {C}}}, v_{\text {max}} \right) , &{} d_i > 0\\ 0, &{} d_i \le 0, \end{array} \right. \end{aligned}$$

where $d_i = \left( \vec {r}_{\text {T},g^*(i,t)} - \vec {r}_i \right) \cdot \vec {e}_i$ is the projection of the AP’s distance from its target position onto its orientation, $v_{\text {max}}$ is a predefined limit for the magnitude of the propulsion velocity and $g^* = \text {argmin}_g {\mathcal {G}}(g)$ is the ideal assignment that minimises ${\mathcal {G}}$. We utilise $v_{\text {max}} = 15\,\sigma /\tau _D$ throughout this work, which has the same order of magnitude as the value used by YB^9^. Analysing equation (2) in detail, it essentially causes APs that randomly point in the directions of their target positions (i.e., $d_i > 0$) to rapidly move to their destinations within the time $\Delta t_{\text {C}}$. APs that point in the wrong direction (i.e., $d_i \le 0$) move only based on Brownian motion until they randomly realign via rotational diffusion and the next update cycle starts. This is depicted in Fig. 1a by the coloured arrows on the APs. For more information, see Ref.^9^.

After the CP is successfully captured, the transport rules can be applied. Here, APs of the HC that randomly point in the predefined transport direction (i.e., along the *x* direction) are used as so-called “transporters”. To be more precise, an AP becomes a transporter if (i) its distance to its assigned target location is smaller than $b = 2 \sigma$, (ii) it possesses more than or equals to $Z = 3$ neighbouring particles with a distance smaller than $1.5 \, \sigma$, and (iii) its orientation randomly lies within a small angular deviation $\Phi _{\text {C}} = \pi / 12$ from the predefined transport direction. If an AP *i* fulfils these conditions, its velocity is set to

3 $$\begin{aligned} v_i = v_{\text {max}}. \end{aligned}$$

Here, $\Phi _{\text {C}}$ is slightly smaller compared to YB’s work^9^, and *Z* is kept constant throughout all our simulations for simplicity (YB vary their *Z* value depending on *N* and $\varepsilon$). The APs that are not chosen as transporters are still moving according to the capture rules and are called “capturers”^9^ (accordingly, during the capture process where the transport rules are not engaged and the feedback-loop only applies the capture rules, all APs are capturers). The assignment, whether an AP is a transporter or a capturer, is again updated after the time $\Delta t_{\text {C}}$. Fig. 1b depicts a schematic representation of the transport rules. Note that we chose the cavity orientation to be parallel to the transport direction to minimise torques on the CP due to temporary uneven distributions of transporters.

If we assume a technical application in the form of microrobots, one normally has a fixed set of micromachines that should perform a maximised number of different tasks. Here, the properties of the active units cannot be changed if different cargo must be transported. With our work, we try to understand the flexibility and limits of hexagonal cage-based interaction rules if one uses the same set of microrobots for all cargo. Therefore, we restrict ourselves to a detailed study of systems where the APs and the CP possess the same diameter $\sigma$ and a detailed parameter study varying the active particle size is outside the scope of this work. Nevertheless, one should keep in mind that changing the size of the APs relative to the CP can strongly influence the following results.

## Capturing rods

We start the discussion of the results by studying the capture process in detail. Before the transport of the cargo makes sense, we have to make sure that a CP is properly enclosed and has a fixed and known location. For this, only the capture rules are applied, and the overall goal is to form a localised HC out of APs that are randomly distributed in the vicinity of the CP. The corresponding BD simulations are performed in units of $\sigma$, $k_B T$, $D_{\text {s}}$ and $\tau _D$. At the start of a simulation, the CP and the APs are randomly placed in a quadratic box with side length $L_{\text {B}} = 50 \, \sigma$, which is centred in a much larger simulation box with a side length of $L_{\text {B}} = 10000 \, \sigma$ (and periodic boundaries). Due to the large simulation box, boundary effects during the formation of the HC are negligible. We perform 500 simulations for each parameter set.

**Figure 2:**
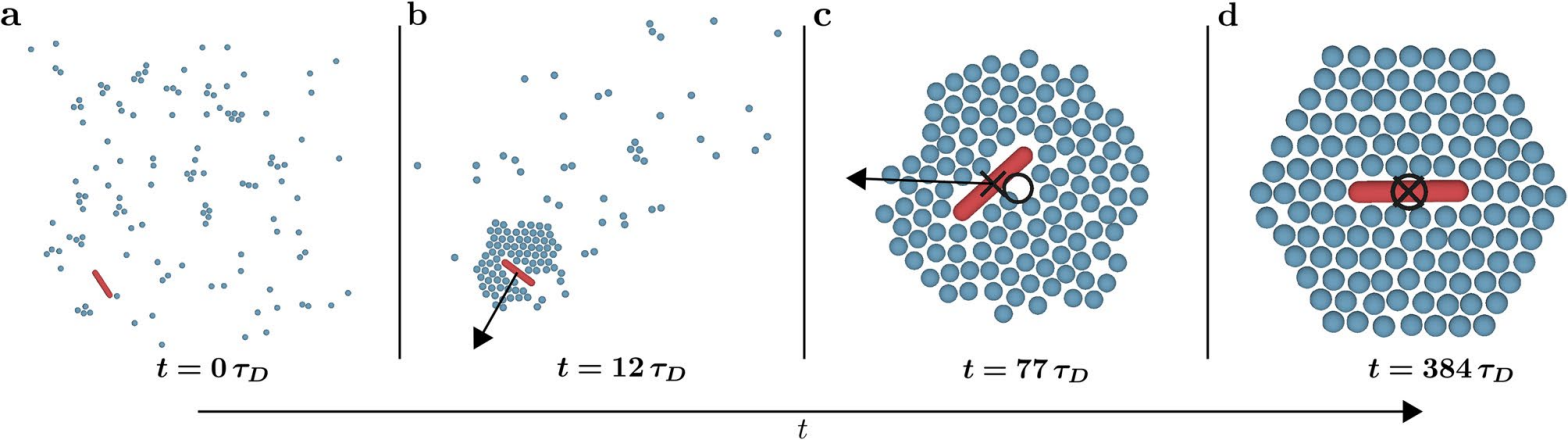
Snapshots of the capture process for a sample system with $q = 5.48$ at $\varepsilon = 5.0\,k_BT$. (**a**) The system starts at random initial conditions. (**b**) After $12\,\tau _D$ (where $\tau _D$ is the Brownian time), several active particles form a cluster around the rod. This cluster performs an active motion (indicated by the black arrow). (**c**) The cluster consists of all active particles at $77\,\tau _D$. However, it does not possess a symmetric shape, and the centre of mass of the active particles (shown by the black circle) and the rod’s centre (shown by the black cross) do not coincide. An active motion (indicated by the black arrow) can be observed. (**d**) After $384\,\tau _D$ the rod is successfully captured. Here, the centre of mass of the active particles and the rod’s centre coincide.

With the adapted capture rules, we can successfully enclose rods in a stable HC (for properly chosen $\varepsilon$ and *q*). The complete capture process (for a sample system with $q = 5.48$ and $\varepsilon = 5.0 \, k_B T$) is presented in Fig. 2 and Supplementary Movie M1. After the capture rules are initiated, the APs quickly gather around the CP and form a cluster. As the particles are randomly distributed at the start of the simulations (see Fig. 2a), this cluster is quite asymmetric at first, and some time is needed before the APs rearrange to the HC (see Fig. 2b and c). In the time the symmetric HC forms (i.e., the time where the APs that are on the “wrong side” concerning their target positions actively push their neighbours while trying to bypass the other particles), the system performs an active motion with distinct ballistic sections and random reorientations without a preferential direction. The resulting random displacements counteract the desired localisation of the system because the particle cluster can travel large distances of multiple $1000 \, \sigma$ in this phase. This is especially visible in Supplementary Movie M1 where the initial asymmetric cluster moves quite rapidly and chaotically before slowing down right before the HC is fully formed (see Fig. 2d).

**Figure 3:**
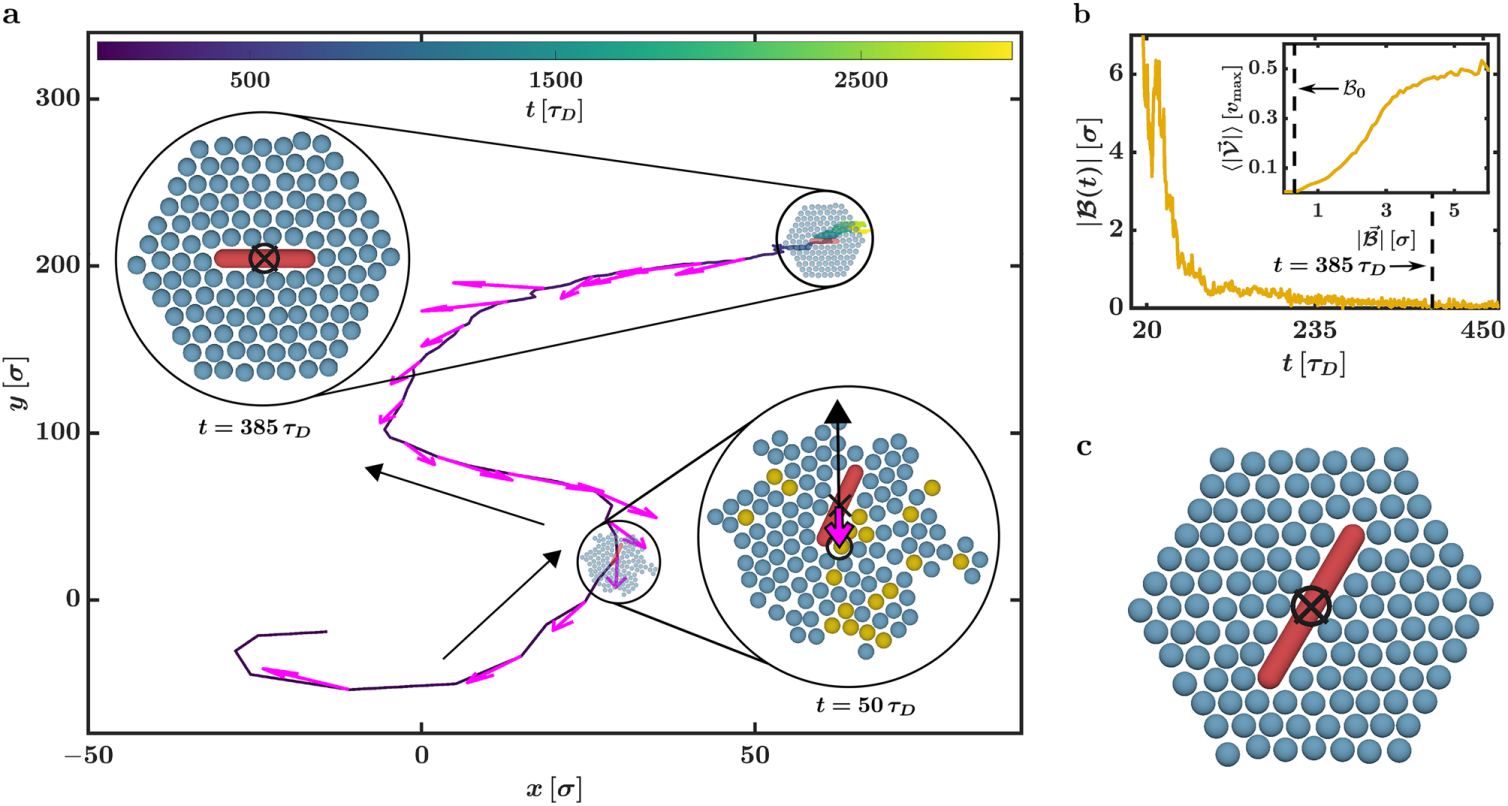
Cargo capture in detail. (**a**) Sample trajectory of a rod’s centre that corresponds to a system with $q = 5.48$ and $\varepsilon = 5.0\,k_BT$. Before the diffusive state is reached, the system performs an active motion for approximately $385\,\tau _D$ (where $\tau _D$ is the Brownian time). Along the trajectory, snapshots of the sample system at $50\,\tau _D$ and $385\,\tau _D$ are shown. For the snapshot at $50\,\tau _D$, the displacement vector $\vec {{\mathcal {B}}}(t)$ between the centre of mass of the active particles and the rod’s centre is depicted via the magenta arrow. Many active particles with a maximum velocity (coloured in orange) are located on one side of the rod pushing it in the opposite direction. The resulting movement direction of the cluster is shown by the black arrow. The thin magenta arrows touching the trajectory mark the displacement vector $\vec {{\mathcal {B}}}(t)$. They are approximately anti-parallel to the overall movement of the cargo particle (black arrows next to the trajectory). (**b**) The corresponding magnitude of the displacement vector $|\vec {{\mathcal {B}}}(t)|$ vanishes with the successful capture after $385\,\tau _D$. The inset is the average magnitude of the rod velocity $\langle |\vec {{\mathcal {V}}}|\rangle$ in dependency of $|\vec {{\mathcal {B}}}|$. A small offset on the $|\vec {{\mathcal {B}}}|$ axis, where a finite displacement vector leads to a vanishing velocity, is found at approximately ${\mathcal {B}}_0 = 0.3\,\sigma$ (dashed line). Here, $\sigma$ is the diameter of the particles. (**c**) A snapshot of an arrangement where a rod with $q = 7.72$ is not parallel to the predefined transport direction (i.e., parallel to the *x*-axis), yet, the diffusive state is reached.

Figure 3a shows the trajectory of the CP centre corresponding to the total simulation time. Propelled by the initially asymmetric AP arrangement, the CP centre performs an active motion until approximately $385\, \tau _D$. Afterwards, it diffuses around a stationary location for multiple $1000 \, \tau _D$. We consider a CP to be successfully captured when such a diffusive state is reached (see Fig. 2d). Note that this transition from an active motion to a diffusive state was not observed in YB’s work^9^ because the APs start at symmetric, predefined initial conditions in their study. The occurrence of the active motion at the start of the capture process is also more dominant for rod-shaped CPs than for spherical CPs, as rotations of the CP constantly push away APs that try to arrange along the target position lattice.

We now try to analyse the transition between the active motion and the diffusive state quantitatively. The displacement vector $\vec {{\mathcal {B}}}(t)$ between the CP centre $\vec {r}_{\text {CP}}(t)$ and the APs’ centre of mass (COM) $\vec {{\mathcal {R}}}(t) = \frac{1}{N} \sum _{j \; \text {is AP}} \vec {r}_j(t)$ is

4 $$\begin{aligned} \vec {{\mathcal {B}}}(t) = \vec {{\mathcal {R}}}(t) - \vec {r}_{\text {CP}}(t). \end{aligned}$$

We find that $\vec {{\mathcal {B}}}(t)$ is an excellent tool to monitor the capture process starting at random initial conditions: After a symmetric HC has formed, the APs’ COM coincides with the CP centre (see Fig. 2d and Supplementary Movie M1, where the circle marks the APs’ COM, and the cross marks the CP centre). Thus, $\vec {{\mathcal {B}}}(t)$ vanishes if the rod is successfully enclosed by the HC and the system reaches the diffusive state. The time evolution of $|\vec {{\mathcal {B}}}(t)|$ corresponding to the trajectory shown in Fig. 3a is depicted in Fig. 3b. The magnitude of the displacement vector $|\vec {{\mathcal {B}}}(t)|$ indeed vanishes after around $385\,\tau _D$ at the same time the capture process ends.

Analysing the orientation of the displacement vector, we find that $\vec {{\mathcal {B}}}(t)$ can efficiently be used to monitor the direction of the active motion before the diffusive state. Due to the asymmetric AP arrangement shortly after the start of the simulations, there is an AP imbalance around the CP (see Fig. 2c). In the direction relative to the CP centre where more APs are located, more particles are still strongly propelled (by the feedback-loop and the capture rules) to reach their target positions (see the configuration at $50\, \tau _D$ in Fig. 3a and Supplementary Movie M2, where all APs with $v_i = v_{\text {max}}$ are marked in orange). These fast-moving APs on one side of the cluster act as the system’s propulsion by pushing it in the direction with fewer APs. On the other hand, the COM of the asymmetric AP cluster is (by its definition) shifted from the current cavity centre, and thus from the CP centre, towards the direction where more APs are located. The displacement vector $\vec {{\mathcal {B}}}(t)$ quantifies this shift. Hence, at a given time *t*, $\vec {{\mathcal {B}}}(t)$ and the system’s movement direction are anti-parallel (see Fig. 3a, where the thick magenta and thin black arrows in the depicted particle configuration for $t = 50\, \tau _D$ mark $\vec {{\mathcal {B}}}(t)$ and the movement direction, respectively). In Supplementary Movie M2, we highlight the current movement direction with a blue arrow, $\vec {{\mathcal {B}}}(t)$ with a magenta arrow and fast-moving APs in orange. While the cluster performs an active motion in this video, we find a one-sided surplus of fast APs and the current movement direction is approximately parallel to $- \vec {{\mathcal {B}}}(t)$. The latter aspect is also visualised in Fig. 3a. Here, the thin magenta arrows along the trajectory indicate the corresponding $\vec {{\mathcal {B}}}(t)$ at different times, and the thin black arrows next to the trajectory show the overall movement direction of the system. We find that the displacement vector $\vec {{\mathcal {B}}}(t)$ is tangential to the trajectory. This verifies that the direction of $- \vec {{\mathcal {B}}}(t)$ corresponds to the system’s movement direction during the capture process. We give a quantitative analysis of this aspect in Supplementary Section S5 of the SI.

In addition to the connections between the movement direction and $\vec {{\mathcal {B}}}(t)$, we also find that the absolute value $|\vec {{\mathcal {B}}}(t)|$ is linked to the magnitude of the CP velocity which we define via

5 $$\begin{aligned} \vec {{\mathcal {V}}}(t) = \frac{\vec {r}_{\text {CP}}(t + \delta t) - \vec {r}_{\text {CP}}(t - \delta t)}{2 \delta t}, \end{aligned}$$

where $\delta t = 0.5 \, \tau _D$ if not stated otherwise. This behaviour is quite clear as a larger AP imbalance lengthens $\vec {{\mathcal {B}}}$. The average absolute value $\langle |\vec {{\mathcal {V}}}| \rangle$ in dependence on $|\vec {{\mathcal {B}}}|$ for a system with $q = 5.48$ and $\varepsilon = 5.0\,k_BT$ is shown in the inset of Fig. 3b (see also Supplementary Section S5 of the SI). If the length of $\vec {{\mathcal {B}}}$ decreases, the corresponding average velocity drops, too. As a result, the CP velocity vanishes for $|\vec {{\mathcal {B}}}| \rightarrow 0$ (i.e., after the APs rearranged to the HC) and the system enters the diffusive state. Due to the intrinsic randomness of the system based on the Brownian fluctuations, there is a small offset where a finite length of $\vec {{\mathcal {B}}}(t)$ still leads to a vanishing mean velocity (marked by the dashed line). This offset in the vicinity of ${\mathcal {B}}_0 = 0.3 \, \sigma$ is of similar magnitude for most of the tested parameter combinations of the aspect ratio *q* and the interaction strength $\varepsilon$.

In contrast to what we showed so far, the capture process can also fail for certain combinations of *q* and $\varepsilon$ or different initial conditions. This is the case when the diffusive state is not reached after the $3000 \, \tau _D$ (scheduled by us for the capture process). For some parameter combinations, the diffusive state is never reached. However, for other choices of *q* and $\varepsilon$, the capture process fails only on rare occasions depending on the initial distribution of the particles. To quantify this, we utilise $\vec {{\mathcal {B}}}(t)$ and ${\mathcal {B}}_0$ to define a criterion of whether a system reaches the diffusive state or not: At the time *t*, a system is assigned to the diffusive state if the ratio $t_{>{\mathcal {B}}_0} / t_{<{\mathcal {B}}_0}$ is below $\delta {\mathcal {B}} = 0.1$. Here, $t_{>{\mathcal {B}}_0}$ is the total time where $|\vec {{\mathcal {B}}}(t)|$ is larger than ${\mathcal {B}}_0 = 0.3 \, \sigma$ in the interval $\left[ t - 100 \, \tau _D, \, t\right]$, and $t_{<{\mathcal {B}}_0}$ is the total time where $|\vec {{\mathcal {B}}}(t)|$ is smaller than ${\mathcal {B}}_0 = 0.3 \, \sigma$ in the interval $\left[ t - 100 \, \tau _D, \, t\right]$. We consider a system “successfully captured” if it is in the diffusive state at $t = 3000\, \tau _D$. Tests showed that this criterion is an efficient tool to find successfully captured CPs. Bear in mind that we highly value a strong localisation of the CP, meaning that our condition of “successfully captured” is stricter than the one of YB^9^.

Note that there are systems with AP arrangements that fulfil this criterion, but the corresponding CP is not captured within the designated cavity parallel to the transport direction. In fact, the CP and the APs can enter a configuration where the rod is aligned with another plane of the triangular HC lattice, but the resulting cage is still symmetric and $\vec {{\mathcal {B}}}(t)$ vanishes (see Fig. 3c where the circle marks the APs’ COM, and the cross marks the CP centre). These configurations correspond to local total energy minima stabilised by the attractions between the APs. Depending on the chosen $\varepsilon$, these minima cannot be overcome by the active motion of the individual APs and, thus, they are meta-stable (tests showed that these AP arrangements stay unchanged for multiple $1000 \, \tau _D$). Systems with such AP arrangements are still considered successfully captured (and it shows that they can be used for cargo transportation). They are also the reason for our relatively large choice of *b* (see the paragraph addressing the transport rules in the Interaction rule section) as for such states, many APs are displaced from their target locations but can easily act as transporters due to the high order in the HC. For more information on these meta-stable states, see Supplementary Section S7 of the SI.

### Geometric and energetic constraints

**Figure 4:**
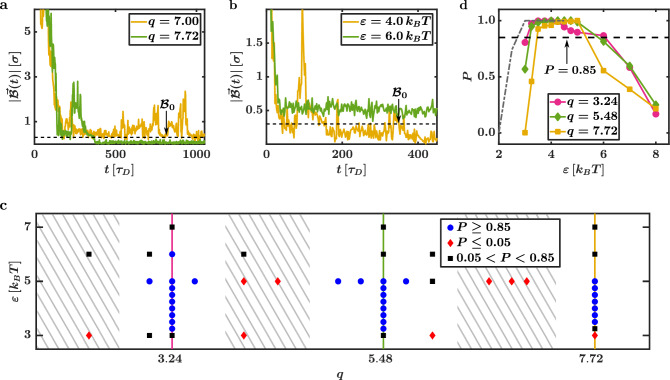
Constraints for a successful capture process. The magnitude of the displacement vector $|\vec {{\mathcal {B}}}(t)|$ for sample systems with the aspect ratios $q = 7.00$ and $q = 7.72$ at the interaction strength $\varepsilon = 5.0\,k_BT$ is depicted in (**a**). In (**b**), $|\vec {{\mathcal {B}}}(t)|$ is shown for sample systems with the aspect ratio $q = 3.24$ at the interaction strengths $\varepsilon = 4.0\,k_BT$ and $\varepsilon = 6.0\,k_BT$. (**c**) Phase diagram depicting the probability *P* that a sample system is captured successfully for varying *q* at different $\varepsilon$. Systems with $P \le 0.05$ are marked with red diamonds, systems with $0.05<P<0.85$ are marked with black squares and systems with $P\ge 0.85$ are marked with blue circles. Periodic *q* intervals with vanishing *P* are hatched in grey. (**d**) Probability *P* for a successful capture process in dependence of $\varepsilon$ for $q = 3.24,\;5.48$ and $q = 7.72$. The black dashed line marks $P = 0.85$. The probability *P* for a spherical cargo particle is shown in grey. The probability *P* vanishes for small $\varepsilon$, sharply rises for intermediate $\varepsilon$, and distinctly drops at large $\varepsilon$. For increasing *q*, the intersection point between *P* and $P=0.85$ is shifted to larger $\varepsilon$.

Analysing $|\vec {{\mathcal {B}}}(t)|$ quantitatively confirms that the success of the capture process strongly depends on *q* and $\varepsilon$. Fig. 4a depicts $|\vec{\mathcal {B}}(t)|$ of sample systems with $q = 7.00$ and $q = 7.72$. Here, $\varepsilon$ is fixed at $\varepsilon = 5.0\,k_BT$. These systems are also shown in Supplementary Movie M3 ($q = 7.00$) and Supplementary Movie M4 ($q = 7.72$). The progression of $|\vec{\mathcal {B}}(t)|$ for systems with the same aspect ratio $q = 3.24$ but two different interactions strengths ($\varepsilon = 4.0$ and $\varepsilon = 6.0$) are depicted in Fig. 4b. The corresponding movies are Supplementary Movie M5 ($\varepsilon = 4.0\, k_ BT$) and Supplementary Movie M6 ($\varepsilon = 6.0 \, k_B T$). We find that, for some parameter combinations ($q=3.24$, $\varepsilon = 4.0\,k_B T$ and $q=7.72$, $\varepsilon = 5.0\,k_B T$) the magnitude of $\vec {{\mathcal {B}}}(t)$ distinctly drops below ${\mathcal {B}}_0$ (i.e., the systems can reach the diffusive state). On the other hand, $|\vec{\mathcal {B}}(t)|$ strongly fluctuates above ${\mathcal {B}}_0$ (i.e., the systems cannot reach the diffusive state) for other combinations ($q=3.24$, $\varepsilon = 6.0\,k_B T$ and $q=7.00$, $\varepsilon = 5.0\,k_B T$). For the depicted systems where $|\vec{\mathcal {B}}(t)|$ stays above ${\mathcal {B}}_0$, additional tests with an extended simulation time showed that this still holds even for $t \ge 5000\,\tau _D$.

To find the specific parameter combinations where the CP can fully be captured, we can use the ${\mathcal {B}}_0$ criterion to introduce the probability *P* that a sample system of a specific parameter combination is captured successfully. We approximate *P* by dividing the number of systems that reached a diffusive state at $t = 3000 \, \tau _D$ by the total number of simulations. In the phase diagram Fig. 4d, the blue circles mark all analysed parameter combinations that correspond to $P \ge 0.85$, and the black squares indicate systems with $0.05< P < 0.85$. Parameter combinations with $P \le 0.05$ are marked by red diamonds.

Most strikingly, the phase diagram shows periodic *q* intervals with vanishing *P*, (which we hatched in grey in the phase diagram to improve readability). It stands out that these intervals are always in the vicinity of aspect ratios *q* that are approximately an even multiple of $a / \sigma$ (or more precisely, they are positioned around aspect ratios that are given by $q = 1 + j \, a / \sigma$, where $j = 1, \, 3, \, 5, \, \dots$ such as $q = 2.12,\;4.36$ and $q = 6.60$). They directly result from the applied capture rules: To form the cavity, target positions in the middle of the HC lattice are removed symmetrically on two sides (see Fig. 1). Therefore, the aspect ratio of the cavity is always an odd multiple of $a / \sigma$ and large empty spaces emerge if a CP with a *q* that is approximately an even multiple of $a / \sigma$ is placed within the cavity. Because of the AP motions and the missing “counter forces” in these empty spaces (which would normally be exerted by the CP), the cavity becomes unstable, and it collapses or does not form in the first place. The APs rather arrange in a cluster with a finite $\vec {{\mathcal {B}}}$, and the capture process fails. This is also visible in the corresponding sample simulation of Supplementary Movie M3 ($q = 7.00$ and $\varepsilon = 5.0\,k_BT$). Here, the APs form an asymmetric cluster after a few $\tau _D$. While they easily organise themselves into a “tight” triangular lattice, the system is unable to form or maintain a symmetric hexagonal configuration. The lasting asymmetry of the cluster propels the system forward (as depicted via the blue arrow in the corresponding movie). This is not the case for a commensurable rod length as depicted in Supplementary Movie M4 ($q = 7.72$ and $\varepsilon = 5.0\,k_BT$). In this sample simulation, although the cluster moves actively at first, the system can fully form the symmetric HC.

While this inability of forming and maintaining the cavity due to the empty spaces appears to be quite intuitive at first, one should note that its occurrence is not clear to begin with because the existence of the HC is based on predefined “social” interaction rules. The formation and the stability of the HC and its cavity are determined by a balance of “artificial” (i.e., the cargo rules executed by the feedback-loop) and “real” physical (i.e., the LJ pair potentials) interactions. As the capture rules do not appear as pair interaction forces between the APs in the calculations, it is difficult to assess for which conditions a stable HC arrangement can be maintained by the feedback-loop. As a consequence, it could generally be possible that the cargo policy could produce a fully formed HC with a stable cavity even in the absence of a CP. In our simulations, however, we find that predefined capture rules are not able to outweigh the physical LJ attractions to maintain the symmetric particle arrangement if empty spaces are present.

Next, we analyse the $\varepsilon$ dependence of *P* by calculating “slices” through the phase diagram (marked by the solid lines in Fig. 4c): The probability *P* in dependence on $\varepsilon$ for the three commensurable aspect ratios $q = 3.24,\; 5.48$ and $q = 7.72$ is depicted in Fig. 4d. For comparison, the grey dashed line indicates the result for a spherical CP (which we also calculated using BD simulations). The general progression of *P* is independent of *q*. It vanishes for small $\varepsilon$ before it sharply rises for intermediate $\varepsilon$ where it reaches values near $P = 1.00$. If $\varepsilon$ is increased further, *P* distinctly drops again.

For $\varepsilon$ near zero, the AP arrangement is only generated via the capture rules. However, due to the translational motion of the particles and the CP’s rotational motions, no stable HC can form as the AP distribution around the CP fluctuates quickly. An example for this is given via Supplementary Movie M7 ($q = 7.72$ and $\varepsilon = 3.0\,k_BT$): Without sufficient interaction strength stabilising the particle arrangements, the capture rules are not able to form and maintain a triangular lattice around the CP (this is in contrast to the sample system depicted in Supplementary Movie M5 [$q = 3.24$ and $\varepsilon = 4.0\,k_BT$] where the formation of the HC benefits from the stronger particle interactions and the CP is captured successfully). The fluctuations of the AP positions result in a non-vanishing (and also fluctuating) $\vec {{\mathcal {B}}}$ and in an active motion of the system, meaning that a localisation of the system fails. At slightly larger $\varepsilon$, the probability for a successful capture process of a spherical CP reaches $P = 1.00$. Contrarily, *P* is still distinctly below $P = 0.85$ for rod-shaped CPs in the same $\varepsilon$ range. Here, rotations of the CP disturb the hexagonal structure of the HC and further destabilise the AP distribution around the CP. As a longer rod “pushes away” more APs while rotating, this effect becomes more pronounced for larger *q* and the points where *P* reaches $P = 0.85$ shift to larger $\varepsilon$ (see the black dashed line in Fig. 4d). Finally, the drop of *P* at large $\varepsilon$ is quite intuitive: For too large $\varepsilon$, the APs’ propulsion velocities are not sufficient to overcome local energy minima of asymmetric AP arrangements. Here, clusters with an AP imbalance and a non-vanishing $\vec {{\mathcal {B}}}$ (i.e., clusters where more APs are located at a specific “side” of the CP) can become (meta-)stable. An example of this is depicted in Supplementary Movie M6 ($q = 3.24$ and $\varepsilon = 6.0\,k_BT$) where the strong attractions between the APs prevent the system from “closing gaps” in the lattice, so a symmetric HC can not fully form. Note that in YB’s work^9^, they argue that the ordered arrangement of the HC mostly results from the capture rules for interaction strengths smaller than $6.0 \, k_B T$, and they indicate that the “real” attractions become strong enough to form a highly ordered structure for $\varepsilon > 6.0 \, k_B T$. This transition point matches well with the range where *P* starts to drop in our studies. Here, the LJ interactions dominate the capture rules that try in vain to give the particle cluster the symmetric HC shape.

As we found strict geometric and energetic constraints, we now restrict ourselves to CPs with commensurable *q* that perfectly fit in a cavity based on an odd multiple of $a / \sigma = 2^{1/6}$ (i.e., $q = 1 + j \, a / \sigma$, where $j = 2, \, 4, \, 6, \, \dots$, and $\varepsilon$ that lead to a probability of $P \ge 0.85$. Altogether, we analyse the aspect ratios $q = 3.24$, $q = 5.48$, and $q = 7.72$ in this work.

### Capture time

**Figure 5:**
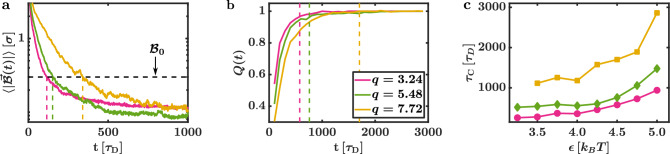
Capture time. The colours are matched between the subfigures. (**a**) Average value of the magnitude of the displacement vector $\langle |\vec {{\mathcal {B}}}(t)|\rangle$ for aspect rations $q = 3.24,\;5.48$ and $q = 7.72$ at an interaction strength $\varepsilon = 4.5\,k_BT$ in a semi-logarithmic plot. The vertical dashed lines indicate the times $\tau _\mathrm{I}$ where $\langle |\vec {{\mathcal {B}}}(t)|\rangle$ falls below ${\mathcal {B}}_0 = 0.3 \, \sigma$ (marked by the dashed black line). Here, $\sigma$ is the diameter of the particles. The time $\tau _\mathrm{I}$ increases with *q*. (**b**) Probability *Q*(*t*) that a successfully captured system has reached a diffusive state at time *t* (for $q = 3.24,\; 5.48$ and $q = 7.72$ at $\varepsilon = 4.5\,k_BT$). The dashed lines indicate the corresponding capture times $\tau _\mathrm{C} = 5\tau _\mathrm{I}$. We find $Q(\tau _{\text {c}}) > 0.90$. (**c**) Capture times $\tau _\mathrm{C}$ in dependence on $\varepsilon$ for $q = 3.24,\;5.48$ and $q = 7.72$. The capture time $\tau _\mathrm{C}$ rises if either $\varepsilon$ or *q* is increased.

Even if these restrictions are met, we still find a strong influence of *q* and $\varepsilon$ on the capture process: The time needed to reach the diffusive state rises with increasing *q* and $\varepsilon$. To estimate the end of the capture process, it is helpful to approximate a capture time $\tau _{\text {c}}$ when the majority of the systems reached the diffusive state. To determine $\tau _{\text {c}}$, we compute the average of the magnitude of the displacement vector $\langle | \vec {{\mathcal {B}}}(t) | \rangle$ regarding all simulations corresponding to a specific parameter combination. The result for systems with $q = 3.24,\;5.48$ and $q = 7.72$ at $\varepsilon = 4.5\,k_BT$ are depicted in Fig. 5a.

In general, $\langle | \vec {{\mathcal {B}}}(t) | \rangle$ is strictly decreasing and all depicted curves fall below ${\mathcal {B}}_0$ before $350 \, \tau _D$ (see the dashed lines in Fig. 5a). The corresponding times $\tau _{\text {I}}$ where $\langle | \vec {{\mathcal {B}}}(t) | \rangle$ reaches ${\mathcal {B}}_0$ for $q = 3.24$, $q = 5.48$, and $q = 7.72$ are $116\,\tau _D$, $152\,\tau _D$ and $341\,\tau _D$, respectively. For all tested parameter sets, we find that after $\tau _{\text {I}}$ approximately $75\%$ of the systems reached the diffusive state. To increase this percentage value even further, we define the capture time by $\tau _{\text {c}} = 5 \tau _{\text {I}}$, and we calculate the probability *Q*(*t*) that a successfully captured system is in the diffusive state after the time *t* to double-check whether our definition of $\tau _{\text {c}}$ is sufficient. Fig. 5b shows *Q*(*t*) for the sample systems. We find that after $\tau _{\text {c}}$ (marked with the dashed lines), more than $90\%$ of the successfully captured systems already reached the diffusive state. Thus, we deem our choice for $\tau _{\text {c}}$ suitable.

Looking at Fig. 5a and b, one can already see that $\tau _{\text {c}}$ depends on the system parameters. Fig. 5c depicts $\tau _{\text {c}}$ in dependence of $\varepsilon$ for a set of *q*. The capture time rises distinctly if either $\varepsilon$ or *q* is increased. The increase in $\tau _{\text {c}}$ with $\varepsilon$ is expected as the rearrangement from the initial cluster to the HC is hindered for stronger AP attractions. In the case of large *q*, the initial AP imbalance around the CP is harder to equalise as fewer APs can reach their assigned targets without bypassing the CP (based on the capture rule design, getting around the CP is rather time-consuming). Furthermore, APs on the way to their target positions act interaction forces and torques on the CP, which further deforms the initial AP cluster with its motion. Longer CPs provide a larger area of collision and longer levers for such interactions.

Note that we also checked if changing ${\mathcal {G}}(g)$ affects $\tau _{\text {c}}$. We find that replacing the sum of distances in equation (1) with the sum of the squared distances slows down the capturing process. However, the dependence on *q* does decrease. See Supplementary Section S8 of the SI.

## Transporting rods

The successfully captured systems can finally be used to start the transport process. In this section, we now apply the transport rules to a system after the capture process has successfully formed a localised HC. We always initiate the transport after $3000\,\tau _D$ making sure to exceed the largest found $\tau _\mathrm{c}$ ($q = 7.72$, $\varepsilon = 5.0\,k_BT$; $\tau _\mathrm{c} = 2863$), and we try to move the corresponding CP along the *x* axis for a total time of $2000\, \tau _D$.

**Figure 6:**
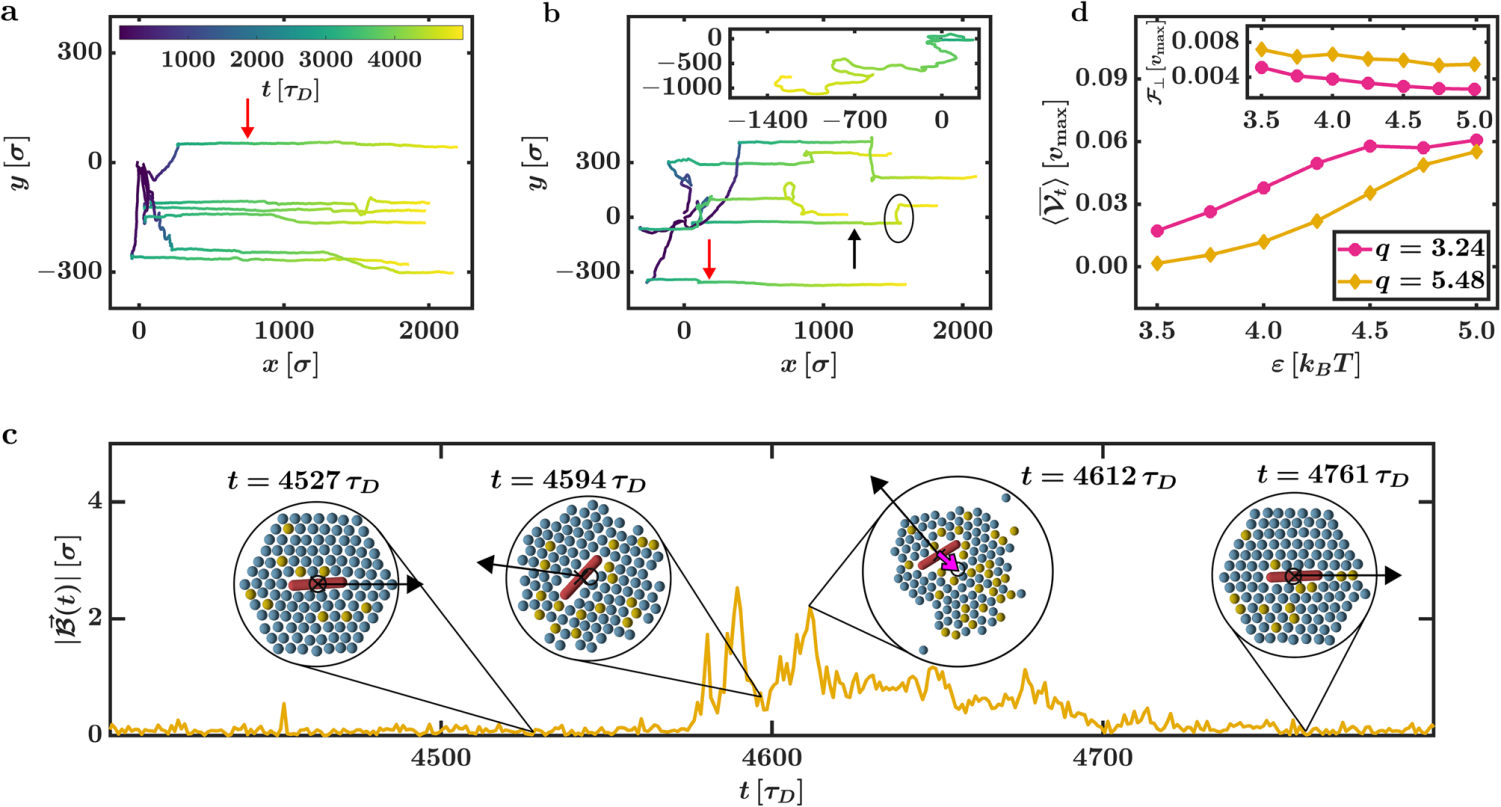
Transport of rods. Transport trajectories of respectively 5 sample systems with the interaction strength $\varepsilon = 5.0\,k_BT$ and the aspect ratios (**a**) $q = 3.24$ and (**b**) $q = 5.48$. The inset shows a trajectory for $q = 7.72$. After the transport is initiated, the rods move parallel to the *x*-axis. While for $q = 3.24$ almost no fluctuations perpendicular to the transport direction can be observed, large fluctuations are present in systems with $q = 5.48$ (for example, in the system marked by the black arrow), and no transport can be achieved for $q = 7.72$. Two systems with vanishing fluctuations are marked by the red arrows. (**c**) Example of an interrupted phase in one of the trajectories (marked by the black ellipse) of (**b**). Snapshots of the system are depicted, and the magnitude of the displacement vector $|\vec {{\mathcal {B}}}(t)|$ is shown. The overall movement of the system is indicated by the black arrows. Active particles with the maximum velocity are marked in orange. When the transport process is interrupted, the hexagonal cage is reshaped into an asymmetric configuration leading to a non-vanishing vector $\vec {{\mathcal {B}}}(t)$. After the cluster again reaches a symmetric configuration, the system starts to precisely move parallel to the transport direction. (**d**) Mean transport velocity $\langle \overline{{\mathcal {V}}_t} \rangle$ in dependence on $\varepsilon$ for $q = 3.24$ and $q = 5.48$. The inset shows the fluctuations ${\mathcal {F}}_{\perp }$ perpendicular to the transport direction. The velocity $\langle \overline{{\mathcal {V}}_t} \rangle$ rises if either $\varepsilon$ is increased or *q* is decreased. The inverse behaviour can be observed for ${\mathcal {F}}_{\perp }$.

Figure 6a and b show multiple CP centre trajectories corresponding to sample systems with $q = 3.24$ and $q = 5.48$ at $\varepsilon = 5.0\,k_BT$. The corresponding movies are Supplementary Movie M8 ($q= 3.24$) and Supplementary Movie M9 ($q = 5.48$). As can be seen through both videos, the transport rules can move rods with different lengths in a predefined transport direction (marked with the black arrow). This is even the case when the CP is not captured so that its orientation is parallel to the transport direction (see Supplementary Movie M8). While spherical CPs can be moved along linear trajectories for sufficient interaction strengths (roughly between $3.0\,k_BT$ and $5.0\,k_BT$)^9^, the transport of rod-shaped CPs appears to be more intermittent: There are multiple interruptions where the system moves in different directions, similar to the asymmetric cluster during the capture process. Their frequency and magnitude increase with *q*, and for $q = 7.72$, the interruptions become dominant enough that the transport fails most of the time (there are sample systems where the transport is successful for longer periods or even throughout the whole simulation, but in a large majority of the simulation, the transport fails). This can be seen in the inset of Fig. 6b or Supplementary Movie M10 ($q = 7.72$ and $\varepsilon = 5.0\,k_BT$), which shows the fluctuating trajectory of a sample system that moves in the wrong direction due to the frequent and lengthy interruptions (in the Supplementary Movie M10, the blue arrow and the black arrow again stand for the current movement direction and the transport direction, respectively).

To understand what causes the interruptions, we analyse particle configurations along the trajectory marked by the black arrow in Fig. 6b. Examples are depicted in Fig. 6c. We find that the HC spontaneously reshapes to an asymmetric cluster, which coincides with the interruptions of the otherwise linear transport process. Afterwards, the APs reform the HC. These rearrangements lead to sections where $| \vec {{\mathcal {B}}} | \gg {\mathcal {B}}_0$ (see Fig. 6c) and the resulting motion parallel to $-\vec {{\mathcal {B}}}$ disrupts the otherwise linear trajectories. The origin of these spontaneous deviations from the HC can be explained by the CP motion within the cavity. CP rotations can introduce internal stress in the HC, which, combined with random fluctuations of the APs, can perturb the AP arrangement. This can lead to an asymmetric cluster with a non-vanishing $| \vec {{\mathcal {B}}} |$. Another important aspect that could lead to the interruptions is the difference between the diffusion coefficients $D^{\parallel }$ and $D^{\perp }$ parallel and perpendicular to the orientation of the CP (see the "Methods" section): If a force $\vec {F}$ is exerted on an overdamped rod with an orientation $\vec {e}$ that is not perfectly parallel or perpendicular to said force, the direction of the resulting rod velocity $\vec {v}$ deviates from the force direction. This is because $\vec {v} \sim [D^{\parallel } \vec {e} \otimes \vec {e} + D^{\perp } (\mathbbm {1} - \vec {e} \otimes \vec {e})] \vec {F}$, where $\otimes$ is the dyadic product, $\mathbbm {1}$ is the unity matrix and we neglect the noise [see equation. (9) of the "Methods" section]. Thus, if a CP is not perfectly parallel to the transport direction, it does not precisely follow the total effective force exerted by the HC to propel the system. This further deforms the HC. As the internal stress due to CP rotations and the difference between $D^{\parallel }$ and $D^{\perp }$ both increase with *q*, deformations of the HC based on these phenomena become more significant for CPs with larger aspect ratios. Finally, it should also be noted that fluctuations in the APs’ positions play a major role in the interruptions of linear transport. During the transport process, the lattice of the HC is also frequently deformed even without the rotations of the CP by fluctuations in the APs’ positions and some APs in the outer layers that momentarily leave their target locations (this was also found for spherical cargo^9^, see also Supplementary Movies M8 and M9). If these fluctuations spontaneously become strong enough that the assignment of the APs to the target locations changes, the interaction rules increase the velocities of the APs and collisions between APs and the CP become more frequent. This again increases the internal stress and the probability of rotations of the CP inside of the cavity.

Note that the capture time of a system with $q = 7.72$ is distinctly higher than $\tau _{\text {c}}$ of the other tested aspect ratios (see Fig. 5c). Hence, the recapture process during the interruptions is too lengthy and the resulting uncontrolled motion yields no net displacement towards the transport direction. This is why we restrict our study further to rods with $q \le 5.48$ (i.e., $q = 3.24$ and $q = 5.48$) which can be transported successfully.

### Transport velocity

A different length of the CP not only decides whether the capture and transport processes are successful, but it also influences the efficiency of the transport: We find that the transport velocity that can be reached decreases with *q* (and increases with $\varepsilon$). Intuitively, the interruptions reduce the average transport velocity as they can lead to movements anti-parallel to the transport directions. Furthermore, fewer APs can work as transporters for increasing *q* because more units are needed to maintain the HC.

The mean transport velocity

6 $$\begin{aligned} \left\langle \overline{{\mathcal {V}}_{\text {t}}}\right\rangle = \frac{1}{t_{\text {t}}} \sum _i \left\langle \vec {{\mathcal {V}}}(t_i) \cdot \vec {n} \delta t \right\rangle, \end{aligned}$$

in dependence on $\varepsilon$ for different *q* is depicted in Fig. 6d. Here, $\vec {n}$ is a unit vector parallel to the transport direction (see Fig. 1b) and $t_{\text {t}} = 2000\,\tau _D$ is the transport process duration. Note that we now introduced two different averages: The angular brackets represent the canonical mean calculated by averaging over different simulations. The bar corresponds to the average regarding $t_{\text {t}}$. As expected, shorter CPs are transported faster. Additionally, we find that $\left\langle \overline{{\mathcal {V}}_{\text {t}}} \right\rangle$ increases for larger $\varepsilon$. This is in line with YB’s work^9^ and results from the higher HC stability at larger $\varepsilon$ (i.e., more APs can act as transporters which increases $\left\langle \overline{{\mathcal {V}}_{\text {t}}}\right\rangle$).

**Figure 7:**
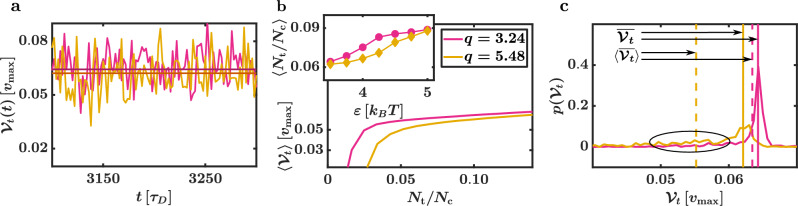
Transport velocity. The colours are matched between the subfigures. (**a**) Transport velocity ${\mathcal {V}}_t(t)$ of the trajectories in Fig. 6a and b marked by the red arrows. The solid lines depict the means $\overline{{\mathcal {V}}_t}$ corresponding to the shown trajectories. The mean transport velocity $\overline{{\mathcal {V}}_t}$ slightly decreases for increasing *q*. (**b**) Average transport velocity $\langle {\mathcal {V}}_t\rangle$ in dependence on the fraction of the numbers of transporters and capturers $N_\mathrm{t}/N_\mathrm{c}$ at interaction strength $\varepsilon = 5.0\,k_BT$ for the aspect ratios $q = 3.24$ and $q = 5.48$ of systems with a linear transport trajectory (i.e., no strong interruptions). Large transport velocities are achieved for large $N_\mathrm{t}/N_\mathrm{c}$ and small aspect ratios. The inset shows the average fraction $\langle N_\mathrm{t}/N_\mathrm{c}\rangle$ in dependence on $\varepsilon$. With increasing *q*, the number of transporter particles decreases. Additionally, longer rods are moved slower by the same number of transporters. (**c**) Probability density of the transport velocity $p({\mathcal {V}}_t)$ for systems with $q = 3.24$ and $q = 5.48$ at $\varepsilon = 5.0\,k_BT$. Here, the solid and dashed lines show the means $\overline{{\mathcal {V}}_t}$ of the sample systems depicted in (**a**) and $\langle \overline{{\mathcal {V}}_t}\rangle$, respectively. An increase in *q* broadens the peak of $p(\overline{{\mathcal {V}}_t})$. Velocities distinctly below $\overline{{\mathcal {V}}_t}$ that correspond to the interruptions lead to a lower overall mean transport velocity $\langle \overline{{\mathcal {V}}_t}\rangle$ for $q = 5.48$.

The fluctuation strength ${\mathcal {F}}_{\perp } = \langle \sqrt{\overline{{\mathcal {V}}^2_{\perp }}}\rangle$ of the CP velocity perpendicular to $\vec {n}$ is depicted in the inset of Fig. 6d. Here, we use ${\mathcal {V}}_{\perp }(t) = \vec {{\mathcal {V}}}(t) \cdot \vec {n}^{\perp }$ and $\vec {n}^{\perp }$ is a unit vector with $\vec {n} \cdot \vec {n}^{\perp } = 0$ (see Fig. 1b). Interestingly, the decrease of ${\mathcal {F}}_{\perp }$ and the increase of $\langle \overline{{\mathcal {V}}_{\text {t}}}\rangle$ are consonant. This indeed suggests a connection between the fluctuations perpendicular to the transport direction (in particular, due to the interruptions) and the value of $\left\langle \overline{{\mathcal {V}}_{\text {t}}}\right\rangle$ that can be reached. Nevertheless, the two linear trajectories marked with red arrows in Fig. 6a and b indicate that the interruptions are not the only factor influencing $\left\langle \overline{{\mathcal {V}}_{\text {t}}}\right\rangle$. Here, the trajectory corresponding to $q = 5.48$ is slightly shorter than its counterpart for $q = 3.24$. The transport velocities ${\mathcal {V}}_t(t) = \vec {{\mathcal {V}}}(t) \cdot \vec {n}$ of these two trajectories are shown in Fig. 7a. The solid lines mark the average of the velocity fluctuations regarding $t_{\text {t}}$ (i.e., the average depicted by the bar and without the angular brackets). Even in the absence of interruptions, the effective mean transport velocity slightly decreases for increasing *q*. This can be understood by analysing the average transport velocity $\langle {\mathcal {V}}_{\text {t}} \rangle$ in dependence on the number of transporters per capturers $N_{\text {t}}/N_{\text {c}}$ for systems that correspond to linear trajectories (see Fig. 7b, $\varepsilon = 5.0 \, k_B T$). To increase the resolution, we decrease $\delta t$ to $\delta t = 0.1\,\tau _D$ for the calculation of $\langle {\mathcal {V}}_{\text {t}} \rangle$. We find that the same number of transporters leads to a lower $\langle {\mathcal {V}}_{\text {t}} \rangle$ for larger *q*. This most likely results from the diffusion coefficients of the CP, which decrease with *q*: An elevation of *q* increases the resistance based on the friction of the CP exerted on the HC. The average number of transporters per capturers $\langle N_{\text {t}}/N_{\text {c}} \rangle$ of systems that correspond to linear trajectories is depicted in the inset of Fig. 7b. Due to the stronger deformations of the HC by longer CPs, more APs are needed as capturers, and consequently, the number of transporters must decrease. While more transporters are needed to transport a longer CP with the same average velocity (Fig. 7b), there are even fewer APs at the disposal that could act as transporters (inset). This explains the *q* dependence of $\overline{{\mathcal {V}}}_t$ even for systems with linear trajectories.

Finally, we have to look into the influences of the interruptions on the mean transport velocity $\left\langle \overline{{\mathcal {V}}}_{\text {t}} \right\rangle$. These influences can be studied by computing the probability density $p(\overline{{\mathcal {V}}_{\text {t}}})$ for a sample system to possess the average transport velocity $\overline{{\mathcal {V}}_{\text {t}}}$. Fig. 7c shows the results for $\varepsilon = 5.0 \, k_B T$. The solid lines in Fig. 7c correspond to the mean velocities $\overline{{\mathcal {V}}_{\text {t}}}$ of the trajectories marked with the red arrows. The dashed lines Fig. 7c are the means $\left\langle \overline{{\mathcal {V}}_{\text {t}}}\right\rangle$ regarding all simulations as shown in Fig. 6d. Firstly, we find that an increase in *q* broadens the peak corresponding to the transport along linear trajectories. The control policy transports longer rods, therefore, less precisely. Secondly, the interruptions lead to a larger percentage of velocities distinctly below the “linear trajectory peak” (see the black ellipse in Fig. 7c). These contributions are the main factor that decreases $\left\langle \overline{{\mathcal {V}}_{\text {t}}}\right\rangle$.

## Discussion

Ideally, the same simple interaction rules can transport arbitrarily shaped cargo reaching from spheres, to rods, to even more complex geometric forms. Here, we confirm that the promising YB rules can be adjusted to handle rods with different aspect ratios, which is a stepping stone towards the transport of arbitrarily shaped microparticles via “swarms” of individual controllable active units. Our study also shows that the shape of the cargo inevitably has a strong impact on the success of interaction rules for cargo transport on the colloidal length scale.

Within the boundaries of the utilised interaction rules, we found that the adjusted YB control policy can, in general, be used to capture and transport rods. Regarding the capture process, however, there are strong geometric and energetic constraints for the cargo particle aspect ratio and the Lennard-Jones interaction strength. If these parameters are correctly chosen, we observed that a successful capture process is characterised by a distinct transition from a chaotic active motion of the system to a stationary diffusive state (starting at random initial conditions). The (capture) time needed to reach this diffusive state increases with the cargo particle aspect ratio. Concerning the transport process, we found that an increase in the aspect ratio hinders the ability to precisely move the cargo particle in a straight line. In fact, if the aspect ratio is chosen too large, the transport is unsuccessful. If the transport of the cargo is possible, an increase in the cargo particle length reduces the transport velocity.

By successfully capturing rods starting at random initial conditions, we showed that no special preparations are needed before activating the feedback-loop. While time-consuming, it is sufficient to randomly utilise the closest active particles to perform the transportation task with the YB rules. Considering an application in the field of microrobots, units could be held in a deactivated, passive state until needed. Then, the units that are randomly the closest to the task can be activated to precisely pinpoint (arbitrarily shaped) colloidal cargo particles via the capture rules and subsequently transport them.

With the application of the displacement vector $\vec {{\mathcal {B}}}$ between the cargo particle centre and the centre of mass of the active particles as well as the introduction of the capture time $\tau _{\text {c}}$, we found a solid framework to describe the properties and the efficiency of the utilised cargo rules. Our definition of the capture time seems to be a promising tool that can be used to benchmark and compare different cargo algorithms to find the most fitting sets for specific tasks. The great advantage of monitoring the swarm behaviour with $\vec {{\mathcal {B}}}$ instead of other quantities, such as the typical effective diffusion coefficients, is that $\vec {{\mathcal {B}}}$ is static in nature: The diffusion coefficients (and corresponding mean square displacements) are dynamic quantities where the whole trajectory in a specific time interval must be known to calculate them. The displacement vector, on the other hand, can be determined for a static particle configuration and at a fixed time to predict the magnitude and directions of subsequent movements. Our method could be applied to various other physical, biological or technical systems in future studies.

Regarding the capture process, we found that it is impossible to capture rods with a length in the vicinity of an even multiple of the lattice constant of the hexagonal cage without further adjustments to the rules. This generally points out an important flaw of all hexagonal cage-based interaction rules: The lattice of the particle arrangement limits the dimensions of the possible cargo particles. Another direct influence of the cargo particle shape on the performance of the interaction rules is the interruptions where the system temporarily leaves the ideal (linear) transport trajectory while performing an active motion. The frequency and magnitude of these interruptions of the transport process also increase with the cargo aspect ratio, and they can even become too strong for the transport to be successful. The interruptions are, in general, linked to the rotational motion of the anisotropic cargo particle. Hence, we predict similar problems when applying a different set of interaction rules.

As the majority of the phenomena and restrictions found are completely geometric in nature, the success rate of the capture and transport process may be increased by changing the size of the active units relative to the dimensions of the cargo particle, or the general particle arrangement. For example, if the diameter of the active units is decreased compared to the diameter of the spherocylinders, the rod can be enclosed by a “tighter” lattice that reduces empty spaces in the cavity and, thus, increase the stability of the configuration (this, however, drastically increases the number of active particles needed to transport the cargo and, consequently, the computational costs). Introducing this additional parameter of a variable active particle diameter may lead to additional restrictions because the size of the smallest possible rod that can be transported via the cargo rules is also limited by the smallest accessible active units. For instance, if we try to smooth out the empty spaces by using active particles with a diameter that is ten times smaller than the cargo diameter, we need perfectly controllable units with a size in the range of $100 \, \text {nm}$ to move a microparticle. Such small programmable active particles may be problematic for laser-based feedback-loop experiments, which usually handle micrometre-sized Janus particles^1–5,8^. Decreasing the diameter of the active particles only slightly compared to the length scale of the cargo particle, on the other hand, could already improve the stability and effectiveness of the cargo capture and transport without distinctly worsening the computational costs. This should be kept in mind while adopting similar rules to other applications.

We should also note that the system and the control policy, in general, possess a large number of different parameters that can be fine-tuned. For example, varying the interaction rule control time, the number of target position layers, the velocity of the active particles, or the criteria that allocate the transporter role will influence the collective motion and the quantitative results. We suggest that each of these parameters is tailored to specific applications of the cargo rules to maximise the success of the concrete tasks. Changing the number of layers, for instance, will increase the mean transport velocity that can be achieved for a fixed interaction strength^9^, but the capture time rises, as well, because more active particles have to find their specific particle location (note that the maximum possible velocity at high attractions only weakly depends on the number of layers, see Ref.^9^). Tests with other numbers of layers also suggest that increasing the number of APs can stabilise the transport of CPs with a larger aspect ratio (see Supplementary Section S4). While increasing the computational cost, decreasing the control time of the feedback-loop can improve the capture process drastically, as Yang and Bevan note in their original work that small control times can avoid collision and blocking phenomena between the active particles^9^ (note that we used a distinctly larger control time compared to Yang and Bevan to save computational cost). Adjusting the general velocity assignment during the capture process [i.e., equation (2)] could also be beneficial because it was designed by Yang and Bevan under the assumption that particle interactions are negligible^9^. Our study shows that this does not hold for the transport of rod-shaped cargo.

Naturally, some of our observations are unique to the utilised interaction rules. Nevertheless, many analysed phenomena are relevant for the transport of non-spherical cargo particles in general. For instance, the rotational movements of the cargo and the non-commensurability regarding a triangular lattice in dense quasi-2D systems will always be additional hindrances.

A hexagonal cage of active particles for the transport of more complexly shaped micro-objects can be implemented by adjusting the cavity. This could lead to further geometric constraints that reduce the applicability, however. Thus, it would be interesting to find additional interaction rules that are less negatively influenced by the particle shape. Many of the recently introduced cargo transport schemes seem promising to handle complex cargo shapes^12,29,30^ and should be tested with a larger variety of differently shaped cargo.

## Methods

In YB’s work^9^, a combination of a repulsive electrostatic potential and an attractive depletion potential is utilised to model the AP attractions. As their fundamental qualitative findings are independent of the concrete AP interactions, we use the generic LJ potential

7 $$\begin{aligned} V_{\varepsilon }(r) = 4 \varepsilon \left[ \left( \frac{\sigma }{r}\right) ^{12} - \left( \frac{\sigma }{r}\right) ^{6}\right] \end{aligned}$$

instead. With this, all AP interactions are determined by a single parameter (for a given diameter $\sigma$), namely the interaction strength $\varepsilon$. To reduce the computational cost, we utilise a cut-off radius of $r_{\text {cut}} = 2.5 \, \sigma$, which is in line with works such as Refs.^31,32^.

The line in the middle of the rectangular section of a spherocylinder that connects the caps is called the line segment. In our work, the CP’s spherocylinder shape is modelled with the Kihara approach^33^, where the shortest distance between the APs’ centres and the line segment of the spherocylinder are utilised to evaluate a predefined pair potential^34^. These distances are calculated as described in Refs.^35–37^. Note that an off-centre interaction between an AP and the spherocylinder also leads to a torque which rotates the CP. This torque is computed using the interaction force and the lever vector defined by the CP centre and the point on the line segment that is connected with the corresponding AP centre via the shortest distance.

In our model, the CP and the APs interact only repulsively. This is achieved by using the WCA potential^26^ for the corresponding pair interactions. It is given by $V_{\text {WCA}}(r) = V_{\varepsilon _{\text {WCA}}}(r) + \varepsilon _{\text {WCA}}$ for $r \le 2^{1/6} \sigma$ and $V_{\text {WCA}}(r) = 0$ otherwise. For simplicity, we use $\varepsilon _{\text {WCA}} = 10 \, k_B T$ throughout all simulations.

We perform 2D BD simulations without hydrodynamic interactions. The integration schemes of the positions $\{\vec {r}_i\}$ and orientations $\{\vec {e}_i\}$ of the particles can be summarised by the relations

8 $$\begin{aligned}&\vec {r}_i(t+\Delta t) = \vec {r}_i(t) + \left[ v_i(t) \Delta t + \frac{D^{\parallel } \Delta t}{k_B T} \left( \vec {F}_i(t) \cdot \vec {e}_i(t) \right) + \sqrt{2 D^{\parallel } \Delta t} R_{i,1}\right] \vec {e}_i(t) \nonumber \\&+ \left[ \frac{D^{\perp } \Delta t}{k_B T} \left( \vec {F}_i(t) \cdot \vec {e}_i^{\perp }(t) \right) + \sqrt{2 D^{\perp } \Delta t} R_{i,2} \right] \vec {e}^{\perp }_i(t) \end{aligned}$$

9 $$\begin{aligned}&\vec {e}_i(t + \Delta t) = \vec {e}_i(t) + \left[ \frac{D^r \Delta t}{k_B T} M_i(t) + \sqrt{2 D^r \Delta t} R_{i,3} \right] \vec {e}_i^{\perp }(t), \end{aligned}$$

where $v_i(t)$ is the magnitude of the active propulsion velocity of the particle *i*, $k_B$ is the Boltzmann constant and *T* is the temperature. The constants $D^{\parallel }$ and $D^{\perp }$ are the translational diffusion coefficients parallel and perpendicular to the corresponding particle orientation, and $D^r$ is the rotational diffusion coefficient. The direction $\vec {e}_i^{\perp }(t)$ is the unit vector perpendicular to the corresponding orientation $\vec {e}_i(t)$. The force $\vec {F}_i(t)$ and the torque $M_i(t)$ result from the interactions and the $R_{i,j}$ are standard normally distributed random numbers. The orientations $\{\vec {e}_i(t+\Delta t)\}$ are normalised after every simulation step.

To describe the motion of the passive rod, we use $v_i(t) = 0$ and the formulas of Ref.^38^

10 $$\begin{aligned} D^\parallel= & {} \frac{k_B T}{2\pi \eta L}\left[ \ln {(q)}-0.1404+\frac{1.034}{q}-\frac{0.228}{q^2}\right] , \end{aligned}$$

11 $$\begin{aligned} D^\perp= & {} \frac{k_B T}{4\pi \eta L}\left[ \ln {(q)}+0.8369+\frac{0.5551}{q}-\frac{0.06066}{q^2}\right] , \end{aligned}$$

12 $$\begin{aligned} D^r= & {} \frac{3k_B T}{\pi \eta L^3}\left[ \ln {(q)}-0.3512+\frac{0.7804}{q}-\frac{0.09801}{q^2}\right] , \end{aligned}$$

for the diffusion coefficients of spherocylinders. For the quasi-2D spherical APs, the diffusion coefficients are set to $D^{\parallel } = D^{\perp } = D_{\text {s}}$ and $D^r = D^r_{\text {s}}$. The magnitude of the propulsion velocities $\{ v_i \}$ at time *t* of the APs are determined with the YB rules. The APs rotate only via passive Brownian motion and, thus, the corresponding scalar torques $\{M_i\}$ vanish.

### Supplementary Information

Supplementary Movie 1.

Supplementary Movie 2.

Supplementary Movie 3.

Supplementary Movie 4.

Supplementary Movie 5.

Supplementary Movie 6.

Supplementary Movie 7.

Supplementary Movie 8.

Supplementary Movie 9.

Supplementary Movie 10.

Supplementary Information.

## Data Availability

The data generated and analysed during the current study are available from the corresponding author upon reasonable request.
