# Supplementary material for: Capture and transport of rod-shaped cargo via programmable active particles
Source: Sci Rep. 2023 Sep 12;13:15071. doi: 10.1038/s41598-023-42119-9 (PMC10497632; doi:10.1038/s41598-023-42119-9)
Supplement: Supplementary file 11 — Supplementary Information. [file 41598_2023_42119_MOESM11_ESM.pdf]

# Supplementary Information to: *Capture and transport of rod-shaped cargo via programmable active particles*

**Philipp Stengele[1,+], Anton Lüders[1,+,*], and Peter Nielaba[1]**

[1]Statistical and Computational Physics, Department of Physics, University of Konstanz, 78457 Konstanz, Germany
[*]anton.lueders@uni-konstanz.de
[+]These authors contributed equally to this work.

## S1 Geometric and energetic constraints for attractive cargo particles

We also checked whether the geometric and energetic constraints regarding the capture process still hold for systems where the cargo particle and the active particles attract each other. For this, we use the Lennard-Jones potential for the interactions between the cargo particle and the active particles instead of the Weeks-Chandler-Andersen potential. The corresponding interaction strength is fixed at $\varepsilon_{\mathrm{CP}-AP} = 10k_BT$ and we utilise a cut-off radius of $r_{\mathrm{cut}} = 2.5\,\sigma$. Note that we use the same $\mathfrak{B}_0 = 0.3\,\sigma$ as in the main article. For each parameter set, we perform 250 simulations. This holds throughout all additional systems studied within the SI.

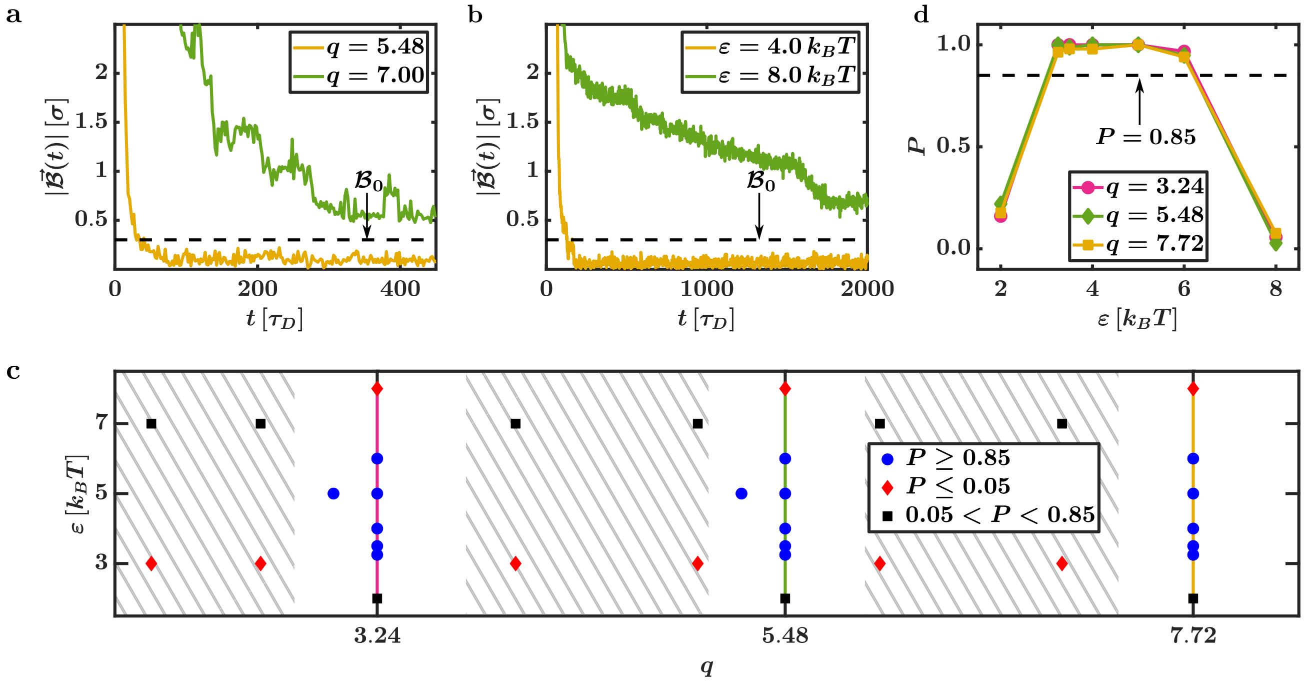


**Figure S1.** Constraints for a successful capture process using the Lennard-Jones potential for the interaction between the cargo particle and the active particles. The displacement vector $|\vec{\mathfrak{B}}(t)|$ is shown for sample systems with (**a**) the aspect ratios $q = 5.48$ and $q = 7.00$ at the interaction strength $\varepsilon = 5.0\,k_BT$, and (**b**) the aspect ratio $q = 3.24$ at the interaction strengths $\varepsilon = 4.0\,k_BT$ and $\varepsilon = 8.0\,k_BT$. (**c**) Phase diagram depicting the probability $P$ for varying $q$ at different $\varepsilon$. Systems with $P \leq 0.05$ are marked with red diamonds, systems with $0.05 < P < 0.85$ are marked with black squares and systems with $P \geq 0.85$ are marked with blue circles. Periodic $q$ intervals with vanishing $P$ are hatched in grey. (**d**) Probability $P$ for successful capture in dependence of $\varepsilon$ for $q = 3.24$, 5.48 and $q = 7.72$. The black dashed line marks $P = 0.85$. The probability $P$ vanishes for small $\varepsilon$ and sharply rises for intermediate $\varepsilon$. For high interaction strengths, $P$ drops again. The progression of $P$ does not depend on $q$.

As for the systems where the cargo particles and the active particles repel each other, we find aspect ratios $q$ that lead to a successful capture process (see Fig. S1a, $q = 5.48$, $\varepsilon = 5.0\,k_BT$) and other $q$ for which the system cannot reach the diffusive

state (see Fig. S1a, $q = 7.00$, $\varepsilon = 5.0, k_B T$) if the interaction strength $\varepsilon$ is fixed. If $q$ is fixed, we find a similar result by varying $\varepsilon$: There are interaction strengths where the system fulfils the $\mathfrak{B}_0$ criterion for a successful capture process (see Fig. S1b, $q = 3.24$, $\varepsilon = 4.0\,k_B T$) and other $\varepsilon$ where the system is not successfully captured (see Fig. S1b, $q = 3.24$, $\varepsilon = 8.0\,k_B T$).

Fig. S1c depicts a phase diagram for the capture success. The blue circles mark all analysed parameter combinations that correspond to $P \geq 0.85$, and the black squares indicate systems with $0.05 < P < 0.85$. Parameter combinations with $P < 0.05$ are marked by red diamonds. Here, $P$ is the probability for a sample system to be in the diffusive state at $t = 3000\,\tau_D$. Some "slices" through the phase diagram for fixed $q$ are shown in Fig. S1d. The general behaviour is identical to the one of the systems in the main article. This means that the geometric and energetic constraints are robust against attractive perturbations in the interaction between the cargo and the active units. However, note that the influence of the aspect ratio becomes negligible and $P$ decays for slightly higher $\varepsilon$ compared to the results of the main article (see Fig. S1d).

## S2 Capture time for attractive cargo particles

Fig. S2 shows the average of the magnitude of the displacement vector $\langle|\vec{\mathfrak{B}}(t)|\rangle$ regarding all 250 simulations corresponding to a specific parameter combination for systems with $q = 3.24$, $q = 5.48$ and $q = 7.72$ in the case of an attraction between the cargo particle and the active particles. The attractive cargo particles interact with the active particle via the Lennard-Jones potential (with $\varepsilon_{CP-AP} = 10.0\,k_B T$ and $r_{\text{cut}} = 2.5\,\sigma$). For consistency, we utilise the same value $\mathfrak{B}_0 = 0.3\,\sigma$ as in the main article. Interestingly, the differences for the various aspect ratios vanish if the cargo particles and the active particles attract each other. All depicted curves intersect the black dashed line marking $\mathfrak{B}_0$ at approximately the same time $\tau_{\text{I}} = 195\,\tau_D$. This indicates that the duration of the capture process is independent of $q$.

We define the capture time for attractive cargo particles also by $\tau_{\text{C}} = 5\tau_{\text{I}}$. The probability $Q(t)$ that a successfully captured system is already in the diffusive state after the time $t$ is depicted in Fig. S2b. The dashed lines correspond to the times $\tau_{\text{C}}$. For attractive particles, we find that more than 85% of the simulations reached the diffusive state at time $\tau_{\text{C}}$. Thus, our definition of the capture time is still sufficient to approximate the duration of the capture process.

The capture time in dependence on $\varepsilon$ for the aspect ratios $q = 3.24$, $q = 5.48$ and $q = 7.72$ is shown in Fig. S2c. The progression of the curves confirms that the duration of the capture process is roughly independent of $q$ if there are attractions between the cargo particle and the active particles.

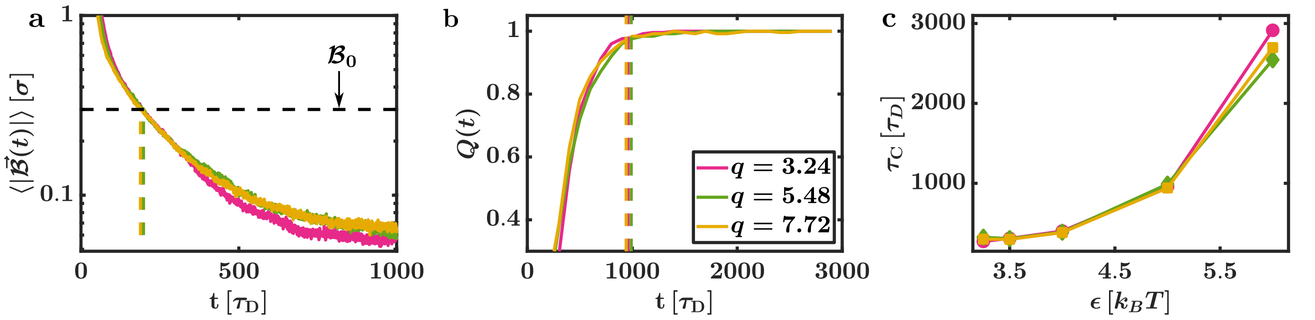


**Figure S2.** Capture time for systems using the Lennard-Jones potential for the interaction between the cargo particle and the active particles. The colours are matched between the subfigures. (**a**) Average value of the magnitude of the displacement vector $\langle|\vec{\mathfrak{B}}(t)|\rangle$ for aspect rations $q = 3.24$, 5.48 and $q = 7.72$ at an interaction strength $\varepsilon = 4.5\,k_B T$ in a semi-logarithmic plot. The vertical dashed lines indicate the time $\tau_{\text{I}}$ where $\langle|\vec{\mathfrak{B}}(t)|\rangle$ falls below $\mathfrak{B}_0 = 0.3\,\sigma$ (marked by the dashed black line). The time $\tau_{\text{I}}$ stays approximately constant for all values of $q$. (**b**) Probability $Q(t)$ that a successfully captured system has reached a diffusive state at time $t$ (for $q = 3.24$, 5.48 and 7.72 at $\varepsilon = 4.5\,k_B T$). The dashed lines indicate the corresponding capture times $\tau_{\text{C}} = 5 \cdot \tau_{\text{I}}$. We find $Q(\tau_{\text{c}}) > 0.85$. (**c**) Capture times $\tau_{\text{C}}$ in dependence on $\varepsilon$ for $q = 3.24$, 5.48 and 7.72. The capture time $\tau_{\text{C}}$ rises if $\varepsilon$ is increased and stays approximately constant for all tested values of $q$.

Strikingly, $\tau_{\text{C}}$ strongly decreases for small $\varepsilon$ particles compared to the results of the main text with the introduction of attractions between the cargo particle and the active particles. Here, the Lennard-Jones potential acting between the cargo particle and the active particles stabilises the inner layer of the hexagonal cage, which accelerates the capture process. For large $\varepsilon$, the capture time for smaller $q$ rises compared to the results of the main article. This is quite intuitive, as the capture rules have to outweigh stronger attractions inside asymmetric clusters to form the hexagonal cage. Nevertheless, our results indicate that an attractive perturbation in the interactions between the cargo particle and the active particles can be beneficial for systems with small $\varepsilon$.

## S3 Example for adjustable active particle interactions in experiments

In modern soft matter set-ups, there are many different ways to tune the interaction between colloidal particles. An important example are quasi-two-dimensional model systems that use magnetic dipole-dipole interactions to modify the concrete particle repulsion[1]. In system of charged colloids, the particular particle interactions can also be tuned by changing the ion concentration in the dispersion medium and, thus, the corresponding screening lengths[2,3].

In Ref. 4 Yang and Bevan identify their numerical implementation of the active particle interactions with entropic potentials. Here, the interaction strength is connected with the properties of the utilised depletion agent[5,6]. Similarly, modifiable colloid interactions can be obtained using critical Casimir forces where the interaction strength can be varied by changing the temperature[7]. In other example systems, the attraction between the colloids can be adjusted using different particle coatings or surface functionalisation[8,9].

Finally, "artificial" interaction rules such as the ones described in Refs. 10 could be beneficial as possible experimental implementations of the cargo transport algorithm need a feedback-loop anyway. Here, cohesion and repulsion parameters can simply be adjusted via computer-based steering signals and particle collision avoidance algorithms.

## S4 Test with a larger number of layers

To understand the influence of the number of layers $m$ (or the number of active particles at our disposal), we performed test simulations with the parameters $q = 7.72$ (aspect ratio), $\varepsilon = 5.0\,k_BT$ (interaction strength), and $m = 9$. For the capture process, we choose a maximum time of $6000\,\tau_D$, and the transport process is conducted for a further $2000\,\tau_D$. We again performed 500 simulations with these parameter combinations.

Increasing $m$ indeed leads to larger capture times (we find $\tau_c = 3845\,\tau_D$). There are even outlier simulations where the diffusive state (i.e., the fully captured, steady state) was not reached after the full $6000\,\tau_D$ simulation time. On the other hand, if the capture process is successful, we find the larger number of layers to be beneficial for cargo transport. With the new number of layers, even rods with an aspect ratio of $q = 7.72$ can easily be transported without larger numbers of interruptions (see the Transporting rods section of the main article). This is quite interesting because the transport of such cargo particles was not possible with a layer number of $m = 7$.

The newly successful transport can be explained by the motion of the active particles close to the rod. With the larger number of layers, we find that the active particles and the cargo particles in the vicinity of the cavity are much more "frozen" in place than for the small number of active particles. Hence, the cargo particle is much less affected by fluctuations in the hexagonal cage's lattice which seems to stabilise the transport.

Our results show that the cargo rules can be adjusted to transport cargo particles with even larger aspect ratios as the ones discussed in the main article. However, this improvement in transport comes with great numerical costs and a distinct increase in the capture time. In practice, one has, therefore, to find a balance between the capture time and the effectiveness of the transport.

## S5 Connection between movement direction and displacement vector

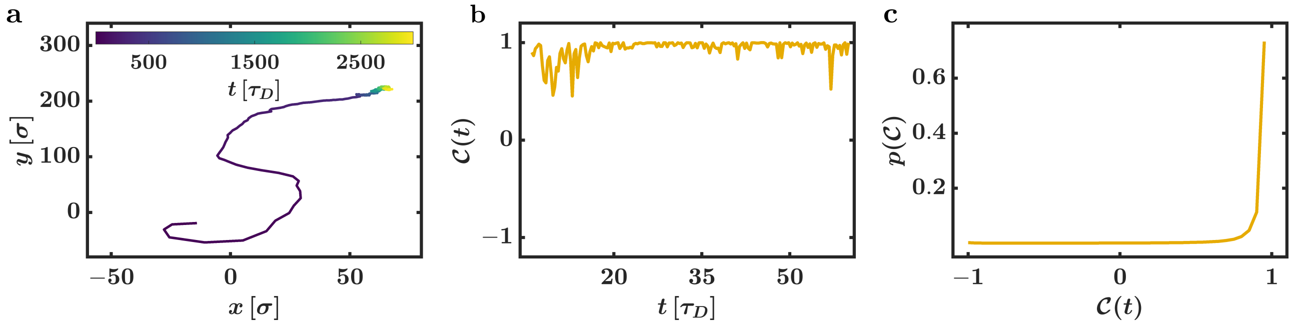


**Figure S3.** Connection between movement direction and displacement vector during the capture process. (**a**) Sample trajectory of a cargo particle centre with $q = 5.48$ at $\varepsilon = 5.0\,k_BT$ up to $3000\,\tau_D$. (**b**) Inner product $\mathrm{C}(t)$ between $\vec{\mathfrak{V}}(t)$ and $-\vec{\mathfrak{B}}$ for the trajectory shown in (a) for the time interval $5\,\tau_D \leq t \leq 60\,\tau_D$. This quantity approximately takes the value $\mathrm{C} = 1$ solidifying that $\vec{\mathfrak{V}}(t)$ and $-\vec{\mathfrak{B}}(t)$ are parallel. (**c**) Probability density $p(\mathrm{C})$ for the quantity C during the active motion averaged over all corresponding simulations. It is sharply distributed around the value $\mathrm{C} = 1$.

Fig. S3b depicts the inner product of the directions of $-\vec{\mathfrak{B}}(t)$ and the cargo particle velocity

$$\mathrm{C}(t) = -\frac{\vec{\mathfrak{B}}(t) \cdot \vec{\mathfrak{V}}(t)}{|\vec{\mathfrak{B}}(t)||\vec{\mathfrak{V}}(t)|} \tag{S1}$$

corresponding to the trajectory of a sample system with $q = 5.48$ and $\varepsilon = 5.0\,k_BT$ shown in Fig. S3a for the time interval $5\,\tau_D \leq t \leq 60\,\tau_D$. While the system moves actively, this quantity approximately takes on the value $\mathrm{C} = 1$. This indicates that the velocity of the cargo particle and the displacement vector are always approximately anti-parallel. The probability density $p(\mathrm{C})$ for the quantity C of the section of the trajectory corresponding to the active motion of the capturing process averaged over all 500 corresponding simulations with $q = 5.48$ and $\varepsilon = 5.0\,k_BT$ is shown in Fig. S3c. The probability density is sharply distributed around the value $\mathrm{C} = 1$, which solidifies that $\vec{\mathfrak{V}}(t)$ and $\vec{\mathfrak{B}}(t)$ are anti-parallel.

## S6 Information to the histogram calculation

The data depicted in Fig. 3b of the main article is calculated by computing the $|\vec{\mathfrak{B}}|$ and $\langle|\vec{\mathfrak{V}}|\rangle$ combinations of $5 \times 10^6$ simulation snapshots collected over all 500 simulations, organising the $|\vec{\mathfrak{B}}|$ values in bins with width 0.1 and averaging the $\langle|\vec{\mathfrak{V}}|\rangle$ values that correspond to the particular $|\vec{\mathfrak{B}}|$ bins. The curves in Fig. 7b are computed with a similar method but only with the systems that are transported along a straight trajectory.

## S7 Diffusive configurations with cargo particles not parallel to the transport direction

A successfully captured system with a cargo particle that is not parallel to the predefined transport direction (here, parallel to the $x$ axis) is shown in Fig. S4a. The parameters are $q = 7.72$ and $\varepsilon = 4.0\,k_BT$.

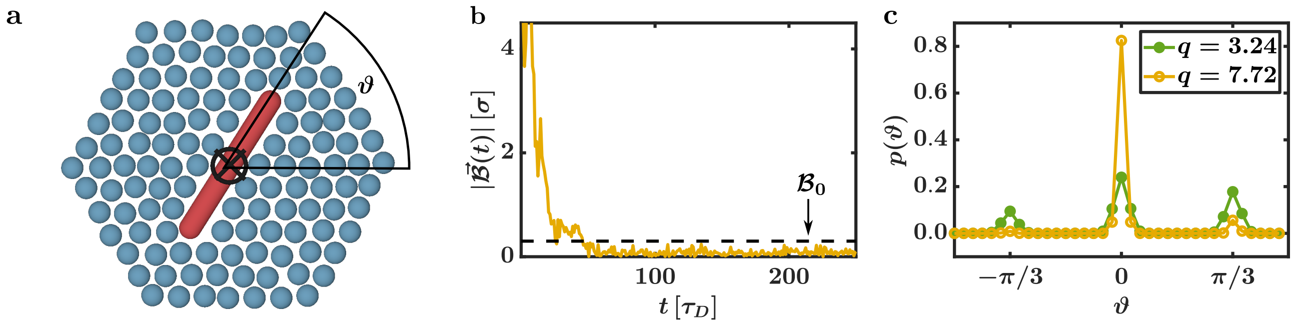


**Figure S4.** Diffusive configurations with cargo particles not parallel to the transport direction. (**a**) A snapshot of an arrangement with $q = 7.72$ and $\varepsilon = 4.0\,k_BT$ where the cargo particle is not parallel to the predefined transport direction, yet, the diffusive state is reached. The angle $\vartheta$ between the cargo particle and the predefined direction is shown in black. (**b**) The absolute displacement vector $|\vec{\mathfrak{B}}(t)|$ of the displayed system (with $q = 7.72$ and $\varepsilon = 4.0\,k_BT$) drops below $\mathfrak{B}_0$. (**c**) Probability density of the angle $\vartheta$ for the aspect ratios $q = 3.24$ and $q = 7.72$ at $\varepsilon = 4.0\,k_BT$. While it is sharply distributed around multiples of $\pi/3$ for $q = 3.24$, the probability density for $q = 7.72$ mostly consists of a larger peak at $\vartheta = 0$ (and vanishing peaks at $\vartheta = \pm\pi/3$).

As indicated by the absolute value of displacement vector $\vec{\mathfrak{B}}$ which drops distinctly below $\mathfrak{B}_0 = 0.3\,\sigma$ (see Fig. S4b), the corresponding system does not move actively but behaves like a system where the cargo particle is captured within a cavity parallel to the transport direction (after the diffusive state is reached). Therefore, such systems can easily be utilised for the transport process. The probability density $p(\vartheta)$ corresponding to the angle $\vartheta$ between the cargo particle and the predefined transport direction is shown in Fig. S4c. It is sharply distributed around multiples of $\pi/3$. These angles correspond to the planes of the triangular lattice. For an angle of $\vartheta = 0$, the probability density reaches its maximum as it is the state desired by the cargo capture rules. There are only small differences between the probability densities of the other allowed angles.

When increasing $q$, the probability density at $\vartheta = 0$ sharply increases while it decreases at the other possible angles (see Fig. S4c). In a system with a longer cargo particle that is rotated outside of the $\vartheta = 0$ direction, more active particles have to be displaced from their intended locations compared to a system with a shorter cargo particle with $\vartheta \neq 0$. Hence, the capture of a cargo particle in a cavity not parallel to the transport direction becomes less favourable if $q$ is increased.

We treat all final configurations corresponding to the different angles $\vartheta$ equally for the capture process. A cargo particle is successfully captured even if the cavity with the cargo particle is not perfectly aligned with the transport direction, but the system is in the diffusive state at $t = 3000\,\tau_D$.

During the intermittent phases of the transport (see the main article, Sec. Transporting rods), the angle $\vartheta$ can change. We find that transitions where the cargo particle is oriented parallel to the transport direction after the non-ballistic phases are frequent. On the other hand, a switch from an orientation parallel to the transport direction to a state with $\vartheta \neq 0$ is rare.

## S8 Capture time for a different cost function

In additional simulations, we also checked if modifying the cost function $\mathcal{G}(g)$ influences the capturing time $\tau_\mathrm{C}$. In particular, we tried the cost function

$$\tilde{\mathcal{G}}(g) = \sum_i \left|\vec{r}_i(t) - \vec{r}_{\mathrm{T},g(i,t)}(t)\right|^2 \tag{S2}$$

which is given by the sum of the squared distances. This function is inspired by the typical methodology applied during linear regression: the method of the least square error. Figs. S5a, b and c showing $\langle|\vec{\mathfrak{B}}(t)|\rangle$, $Q(t)$ and $\tau_\mathrm{C}$ for the simulations with $\tilde{\mathcal{G}}(g)$ can directly be compared with Fig. 5 of the main article. Note that we perform again 250 simulations per parameter set.

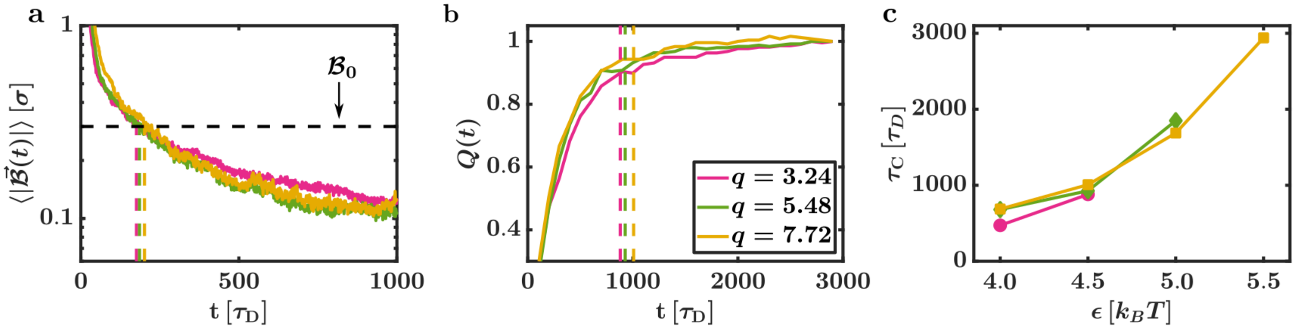


**Figure S5.** Capture time for systems with a different cost function. The colours are matched between the subfigures. (**a**) Average value of the magnitude of the displacement vector $\langle|\vec{\mathfrak{B}}(t)|\rangle$ for aspect rations $q = 3.24$, 5.48 and $q = 7.72$ at an interaction strength $\varepsilon = 4.5\,k_BT$ in a semi-logarithmic plot. The vertical lines indicate the time $\tau_\mathrm{I}$ where $\langle|\vec{\mathfrak{B}}(t)|\rangle$ falls below $\mathfrak{B}_0 = 0.3\,\sigma$ (marked by the dashed black line). (**b**) Probability $Q(t)$ that a successfully captured system has reached a diffusive state at time $t$ (for $q = 3.24$, 5.48 and $q = 7.72$ at $\varepsilon = 4.5\,k_BT$). The dashed lines indicate the corresponding capture times $\tau_\mathrm{C} = 5\tau_\mathrm{I}$. We find $Q(\tau_\mathrm{c}) > 0.85$. (**c**) Capture times $\tau_\mathrm{C}$ in dependence on $\varepsilon$ for $q = 3.24$, 5.48 and 7.72. The slope increases compared to the results of the main article.

We find that replacing $\mathcal{G}(g)$ with $\tilde{\mathcal{G}}(g)$ does indeed affect the general behaviour of the capturing process: Firstly, the slope of the capturing time regarding $\varepsilon$ seems to increase for the new cost function and small $q$. This means that the capturing process needs, in general, more time. Secondly, the discrepancy between the different aspect ratios vanishes. This is quite interesting and suggests that some of the shape dependence of the cargo rules can be compensated by varying the cost function. Also, the capture time for large $q$ and large $\varepsilon$ seems to decrease. Here, further studies are needed which are outside of the scope of this work.

## Information on supplementary movies

To visualise the results of this work, we provide various movies depicting the capture and transport of rod-shaped cargo. Throughout these movies, the blue arrow in the upper right corner depicts the current movement direction of the system. In more detail, it depicts the average displacement of the cargo particle centre regarding the last five movie frames. The blue arrow disappears when the length of the mean displacement of the cargo centre regarding the last five movie frames is smaller than $0.45\,\sigma$. Note that the movement direction of the first five frames is not shown as, here, the average of the last five does not exist. If the transport rules are applied to the system, an additional black arrow indicating the predefined transport direction is shown. The numbers in the movies' captions are the time in units of the Brownian time $\tau_D$. The movies M1 to M7 depict systems where only the capture rules are applied. In M8 to M10, the transport rules are engaged after a few $\tau_D$.

- **M1:** Successful capture process of a sample system with $q = 5.48$ and $\varepsilon = 5.0\,k_BT$. Here, only the cluster rules are engaged. Starting from random initial conditions, an asymmetric cluster forms first. This cluster performs an active motion with distinct ballistic sections and random reorientation counteracting the localisation of the system. After the hexagonal cage has fully formed, the cargo particle is successfully captured and the active motion stops.

- **M2:** Active motion during the capture process of a system with $q = 5.48$ and $\varepsilon = 5.0\,k_BT$. Again, only the cluster rules are engaged. The strongly propelled active particles are marked with an orange colour. While the cluster performs an active motion in the video, we find a one-sided surplus of fast APs and the current movement direction is approximately parallel to $-\vec{\mathfrak{B}}(t)$.
- **M3:** Unsuccessful capture process of a sample system with $q = 7.00$ and $\varepsilon = 5.0\,k_BT$ (only the cluster rules are used). While the APs easily organise themselves into a "tight" triangular lattice, the system is unable to form or maintain a symmetric hexagonal form. The lasting asymmetry of the cluster propels the system forward.
- **M4:** Successful capture process of a sample system with $q = 7.72$ and $\varepsilon = 5.0\,k_BT$, where only the cluster rules are applied. In this simulation, the cluster moves also actively but the system can fully form the symmetric HC.
- **M5:** Successful capture process of a sample system with $q = 3.24$ and $\varepsilon = 4.0\,k_BT$ (only the cluster rules are engaged). The formation of the HC benefits from the stronger particle interactions and the CP is captured successfully.
- **M6:** Unsuccessful capture process of a sample system with $q = 3.24$ and $\varepsilon = 6.0\,k_BT$, where only the cluster rules are engaged. Here, the strong attractions between the AP prevent the system from "closing gaps" in the lattice and, thus, a symmetric HC can not fully form.
- **M7:** Sample system with $q = 7.72$ and $\varepsilon = 3.0\,k_BT$. Still, only the cluster rules are engaged. Without sufficient interaction strength stabilising the particle arrangements, the capture rules are not able to form and maintain a triangular lattice around the CP.
- **M8:** Transport process of a sample system with $q = 3.24$ and $\varepsilon = 5.0\,k_BT$. Here, the transport rules are applied after a few $\tau_D$. Note that the active particles that are not transporters move according to the capture rules. We find that rods can be successfully transported via the transport rules (even if they are not captured parallel to the predefined transport direction).
- **M9:** Transport process of a sample system with $q = 5.48$ and $\varepsilon = 5.0\,k_BT$, where the transport rules are applied after a few $\tau_D$. Again, keep in mind that the active particles that are not transporters move according to the capture rules. The transport rules can be utilised to move rods of different lengths. Here, a sample system with $q = 5.48$ is depicted.
- **M10:** Unsuccessful transport of a sample system with $q = 7.72$ and $\varepsilon = 5.0\,k_BT$ (the transport rules are applied after a few $\tau_D$). The video shows the fluctuating trajectory of a sample system that moves in the wrong direction due to frequent and lengthy interruptions.

## References


1. Promislow, J. H. E., Gast, A. P. & Fermigier, M. Aggregation kinetics of paramagnetic colloidal particles. *The J. Chem. Phys.* **102**, 5492–5498; 10.1063/1.469278 (1995).
2. Gutsche, C., Keyser, U. F., Kegler, K., Kremer, F. & Linse, P. Forces between single pairs of charged colloids in aqueous salt solutions. *Phys. Rev. E* **76**, 031403; 10.1103/PhysRevE.76.031403 (2007).
3. Tavares, F., Bratko, D. & Prausnitz, J. The role of salt–macroion van der Waals interactions in the colloid–colloid potential of mean force. *Curr. Opin. Colloid & Interface Sci.* **9**, 81–86; https://doi.org/10.1016/j.cocis.2004.05.008 (2004).
4. Yang, Y. & Bevan, M. A. Cargo capture and transport by colloidal swarms. *Sci. Adv.* **6**, eaay7679; 10.1126/sciadv.aay7679 (2020).
5. Rudhardt, D., Bechinger, C. & Leiderer, P. Direct measurement of depletion potentials in mixtures of colloids and nonionic polymers. *Phys. Rev. Lett.* **81**, 1330–1333; 10.1103/PhysRevLett.81.1330 (1998).
6. Helden, L., Koenderink, G. H., Leiderer, P. & Bechinger, C. Depletion potentials induced by charged colloidal rods. *Langmuir* **20**, 5662–5665; 10.1021/la0495711 (2004).
7. Helden, L. *et al.* Critical casimir interactions of colloids in micellar critical solutions. *Soft Matter* **17**, 2737–2741; 10.1039/D0SM02021D (2021).
8. Hong, L., Cacciuto, A., Luijten, E. & Granick, S. Clusters of amphiphilic colloidal spheres. *Langmuir* **24**, 621–625; 10.1021/la7030818 (2008).
9. Geerts, N. & Eiser, E. DNA-functionalized colloids: Physical properties and applications. *Soft Matter* **6**, 4647–4660; 10.1039/C001603A (2010).

**10.** Knippenberg, T., Lüders, A., Lozano, C., Nielaba, P. & Bechinger, C. Role of cohesion in the flow of active particles through bottlenecks. *Sci. Rep.* **12**, 11525; 10.1038/s41598-022-15577-w (2022).
